# Kinetic Properties of Microbial Exoenzymes Vary With Soil Depth but Have Similar Temperature Sensitivities Through the Soil Profile

**DOI:** 10.3389/fmicb.2021.735282

**Published:** 2021-11-30

**Authors:** Ricardo J. Eloy Alves, Ileana A. Callejas, Gianna L. Marschmann, Maria Mooshammer, Hans W. Singh, Bizuayehu Whitney, Margaret S. Torn, Eoin L. Brodie

**Affiliations:** ^1^Climate and Ecosystem Sciences Division, Lawrence Berkeley National Laboratory, Berkeley, CA, United States; ^2^Department of Civil and Environmental Engineering, University of California, Los Angeles, Los Angeles, CA, United States; ^3^Department of Environmental Science, Policy, and Management, University of California, Berkeley, Berkeley, CA, United States; ^4^Department of Molecular and Cell Biology, University of California, Berkeley, Berkeley, CA, United States; ^5^Department of Plant and Microbial Biology, University of California, Berkeley, Berkeley, CA, United States; ^6^Energy and Resources Group, University of California, Berkeley, Berkeley, CA, United States

**Keywords:** extracellular enzymes, soil, subsoil, temperature sensitivity, Michaelis-Menten, Arrhenius, macromolecular rate theory, enzyme kinetics

## Abstract

Current knowledge of the mechanisms driving soil organic matter (SOM) turnover and responses to warming is mainly limited to surface soils, although over 50% of global soil carbon is contained in subsoils. Deep soils have different physicochemical properties, nutrient inputs, and microbiomes, which may harbor distinct functional traits and lead to different SOM dynamics and temperature responses. We hypothesized that kinetic and thermal properties of soil exoenzymes, which mediate SOM depolymerization, vary with soil depth, reflecting microbial adaptation to distinct substrate and temperature regimes. We determined the Michaelis-Menten (MM) kinetics of three ubiquitous enzymes involved in carbon (C), nitrogen (N) and phosphorus (P) acquisition at six soil depths down to 90 cm at a temperate forest, and their temperature sensitivity based on Arrhenius/*Q*_10_ and Macromolecular Rate Theory (MMRT) models over six temperatures between 4–50°C. Maximal enzyme velocity (*V*_max_) decreased strongly with depth for all enzymes, both on a dry soil mass and a microbial biomass C basis, whereas their affinities increased, indicating adaptation to lower substrate availability. Surprisingly, microbial biomass-specific catalytic efficiencies also decreased with depth, except for the P-acquiring enzyme, indicating distinct nutrient demands at depth relative to microbial abundance. These results suggested that deep soil microbiomes encode enzymes with intrinsically lower turnover and/or produce less enzymes per cell, reflecting distinct life strategies. The relative kinetics between different enzymes also varied with depth, suggesting an increase in relative P demand with depth, or that phosphatases may be involved in C acquisition. *V*_max_ and catalytic efficiency increased consistently with temperature for all enzymes, leading to overall higher SOM-decomposition potential, but enzyme temperature sensitivity was similar at all depths and between enzymes, based on both Arrhenius/*Q*_10_ and MMRT models. In a few cases, however, temperature affected differently the kinetic properties of distinct enzymes at discrete depths, suggesting that it may alter the relative depolymerization of different compounds. We show that soil exoenzyme kinetics may reflect intrinsic traits of microbiomes adapted to distinct soil depths, although their temperature sensitivity is remarkably uniform. These results improve our understanding of critical mechanisms underlying SOM dynamics and responses to changing temperatures through the soil profile.

## Introduction

Soils are estimated to contain ∼3,000 Gt carbon (C), which is more than all C in the atmosphere and in living biomass combined (Köchy et al., 2015). The dynamics of the large soil C reservoir is sensitive to climate change, and C losses as carbon dioxide (CO_2_) are expected to become a major positive feedback to global warming through increased soil organic matter (SOM) decomposition (Crowther et al., 2016; Van Gestel et al., 2018). An estimated 55 ± 50 Gt C may be lost globally from just 1°C warming of the upper 10 cm of soil alone (Crowther et al., 2016). While current model predictions of C dynamics and responses to climate change are largely based on surface soils (Trumbore, 2009; Crowther et al., 2016; Van Gestel et al., 2018), soils below 20 cm contain up to 50% of the global soil C budget within the top 1 m of soil (Jobbágy and Jackson, 2000; Balesdent et al., 2018). These subsoils are predicted to warm at rates similar to those of surface soils (Soong et al., 2020), and recent *in situ* deep soil warming experiments have shown uniform warming responses down to 100–120 cm depth leading to soil C losses at least three times higher than those estimated based on surface soils alone (Hicks Pries et al., 2017; Hanson et al., 2020; Nottingham et al., 2020; Soong et al., 2021). Despite these observations, relatively little is known about the microbial mechanisms and interactions mediating SOM turnover and CO_2_ emissions, and their responses to environmental changes in subsoils (Rumpel and Kögel-Knabner, 2011; Gross and Harrison, 2019), which are essential to improve predictions of SOM dynamics in response to warming.

Soil physicochemical properties and environmental conditions, such as nutrient inputs, temperature, moisture, mineralogy, and organic matter composition vary markedly with depth, creating distinct environments for the microbial processes that mediate SOM transformations (Trumbore, 2000; Blume et al., 2002; Fierer et al., 2003a; Salomé et al., 2010; Rumpel and Kögel-Knabner, 2011; Jones et al., 2018). The rate-limiting steps in SOM decomposition are primarily catalyzed by microbial exoenzymes, which depolymerize plant and microbial residues into lower molecular weight compounds that are assimilated by both plants and microbes (Davidson and Janssens, 2006; Burns et al., 2013). The kinetic and thermal properties of exoenzymes are therefore fundamental determinants of SOM turnover, nutrient availability, soil C stability, and greenhouse gas emissions, as well as their responses to environmental changes (Davidson and Janssens, 2006; Wallenstein et al., 2011; Sinsabaugh and Shah, 2012; Chen et al., 2018). In addition to the large diversity of exoenzymes targeting different organic compounds, evolutionarily distinct exoenzymes that catalyze the same reactions (i.e., isozymes) can vary widely in their kinetic properties, namely their catalytic rate constant, or turnover number (*k*_cat_), and related maximal reaction velocity (*V*_max_), their Michaelis constant (*K*_m_), which is inversely proportional to their affinity for the substrate, and their catalytic efficiency (*k*_cat_*/K*_m_) (Khalili et al., 2011; Nannipieri et al., 2012; Sinsabaugh and Shah, 2012; Tischer et al., 2015). These properties constitute microbial evolutionary adaptations and trade-offs related to resource supply and demand, as well as other environmental constraints, such as temperature and pH, associated with distinct ecological niches (Allison et al., 2011; Sinsabaugh and Shah, 2012; Ho et al., 2017; Malik et al., 2020). In soils, for example, exoenzyme kinetics have been shown to reflect variation in nutrient availability, pH, climate, and plant root proximity (Baker and Allison, 2017; Zhang et al., 2018; Puissant et al., 2019; Tian et al., 2020). Temperature is also a major factor controlling microbial community assembly, growth and functionality (Allison and Treseder, 2008; Bradford, 2013; Cavicchioli et al., 2019; Lax et al., 2020), and warming has been shown to change the abundance of diverse taxa and functional groups through the soil profile (Jiang et al., 2020; Dove et al., 2021). Moreover, temperatures of optimal enzyme activity are broadly correlated with the optimal growth temperatures of their organisms, as well as with the frequency of specific metabolic pathways, reflecting a concerted evolutionary adaptation to temperature and associated selective pressures (Somero, 2004; Engqvist, 2018). Therefore, variation in substrate and temperature regimes through the soil profile is likely to select for microbiomes producing enzymes with distinct kinetic and thermal properties, which may impose depth-dependent constraints on SOM turnover and responses to warming (Allison and Treseder, 2008; Carrillo et al., 2018; Cavicchioli et al., 2019; Isobe et al., 2019; Nunan et al., 2020; Xu et al., 2021). Microbial community composition and functional potential have indeed been shown to vary strongly with soil depth, reflecting selective adaptation to distinct niches (Blume et al., 2002; Fierer et al., 2003b; Hansel et al., 2008; Hartmann et al., 2009; Eilers et al., 2012; Turner et al., 2017; Jiao et al., 2018; Brewer et al., 2019; Diamond et al., 2019; Liu et al., 2019; Yan et al., 2019; Dove et al., 2021; Zosso et al., 2021). At the same time, exoenzyme activities in nature are dependent on multiple factors that can directly or indirectly modulate their kinetics, thermodynamics, and expression, beyond the intrinsic traits of the microbiome and the enzymes they encode. In particular, microbe-plant interactions, soil properties, and environmental conditions all affect enzyme expression, turnover, mobility, and substrate accessibility (Davidson and Janssens, 2006; Wallenstein et al., 2011; Bradford, 2013; Burns et al., 2013; Tang and Riley, 2019). Consequently, the effective kinetics of mixed exoenzyme pools in complex environments are emergent properties that reflect not only the summation of traits from distinct isozymes and organisms, but also direct and indirect interactions between enzymes and the environment (Davidson and Janssens, 2006; Sinsabaugh and Shah, 2012; Burns et al., 2013).

Given the critical role of exoenzymes in soil C stability and CO_2_ emissions, their activities and environmental controls have been extensively studied in the context of warming and other environmental changes, as indicators of SOM decomposition activity and nutrient availability (Allison et al., 2011; Sinsabaugh and Shah, 2012; Burns et al., 2013). Despite efforts to also integrate kinetic and thermal properties of exoenzymes to better understand the mechanisms of SOM turnover in response to warming (Wallenstein et al., 2011; German et al., 2012; Burns et al., 2013; Razavi et al., 2015, 2016; Alster et al., 2016a,^2020^; Loeppmann et al., 2016a; Allison et al., 2018), most studies have focused on one kinetic property (i.e., *V*_max_) and/or on surface soils. Several studies have investigated exoenzyme activities through the soil profile (Taylor et al., 2002; Venkatesan and Senthurpandian, 2006; Gelsomino and Azzellino, 2011; Kramer et al., 2013; Schnecker et al., 2014, 2015; Stone et al., 2014; Loeppmann et al., 2016a; Jing et al., 2017; Darby et al., 2020; Dove et al., 2020). However, nearly all of these studies, possibly with just one exception (Loeppmann et al., 2016a), have relied on enzyme activity assays based on single substrate concentrations and have not experimentally determined the Michaelis-Menten (MM) kinetics required to accurately estimate *V*_max_, as well as *K*_m_ and catalytic efficiency, which cannot be otherwise inferred. While both approaches share the same technical limitations and must be interpreted in the context of complex enzyme pools and environmental samples, assays using single substrate concentrations are also prone to underestimate the full enzyme activity potential (i.e., *V*_max_), as substrate may be below the enzyme saturation point, or exceed it to the point of inhibition (Wallenstein et al., 2011). Soil temperature regimes are well-known to vary with depth, as heat diffusion is dampened through the soil profile, leading to narrower temperature ranges in deeper soils and preventing them from reaching the same temperature extremes as those at the surface (Al-Kaisi et al., 2017). However, the temperature sensitivity of soil exoenzymes produced by microbes potentially adapted to these distinct depth-dependent temperature regimes has rarely been characterized (Steinweg et al., 2013). Moreover, studies that determined both the MM kinetics of exoenzymes and their direct temperature sensitivity are scarce, even for surface soils (Razavi et al., 2015, 2016; Allison et al., 2018).

The temperature sensitivity of soil exoenzymes and other biogeochemical processes has been typically determined based on the linear Arrhenius model and related *Q*_10_ coefficient, which represents a simple empirical metric expressing variation in activity rates at every 10°C change in temperature (Alster et al., 2020). However, it has been argued that the *Q*_10_ coefficient may not reliably represent soil biological processes, as it lacks a biological and mechanistic basis, and does not capture the unimodality of typical enzyme reactions (Hobbs et al., 2013; Alster et al., 2020). These caveats possibly explain the frequent inability of *Q*_10_-values to describe observed temperature responses of soil biological processes, and lack of comparability between studies (Alster et al., 2020). Macromolecular Rate Theory (MMRT) has been recently proposed as a more realistic model of enzyme temperature sensitivity based on thermodynamics and the change in heat capacity associated with enzyme catalysis, which accounts for declines in enzyme activity below thermal denaturation temperatures (Hobbs et al., 2013). MMRT can thus appropriately capture the unimodal behavior of enzyme response to temperature, and describes temperature sensitivity as comprising three fundamental components: temperature optimum (*T*_opt_), the temperature at which reaction rates are maximal; point of maximum temperature sensitivity (TS_max_), the temperature at which reaction rates change the most; and change in heat capacity (Δ*C*_p_^‡^), which describes the degree of curvature of the parabolic response of reaction rates to temperature (Alster et al., 2020). A limited number of studies have applied MMRT to soil biological activities, including exoenzymes in soils and cultures of soil microbes, where it could describe temperature responses more coherently than Arrhenius models and provide more realistic interpretations of temperature sensitivity (Schipper et al., 2014; Alster et al., 2016a,b, 2018; Robinson et al., 2017). However, to our knowledge, MMRT has never been used to investigate the temperature sensitivity of exoenzymes over the whole soil profile.

Different soil models have been developed to represent exoenzyme kinetics, thermodynamics, ecological stoichiometry, enzyme diffusion, and interactions with environmental factors (German et al., 2012; Sinsabaugh and Shah, 2012; Sulman et al., 2014; Wieder et al., 2014; Tang and Riley, 2015, 2019; Wang et al., 2015; Wang and Allison, 2019). However, these processes have only recently started to be incorporated into depth-resolved soil biogeochemical models (Dwivedi et al., 2019; Wang et al., 2021), are rarely considered in fully coupled ecosystem scale models (Grant, 2013; Pasut et al., 2021), and are entirely unrepresented in current Earth system models. Moreover, exoenzyme kinetics, when included in depth-resolved models, are represented as a function of microbial biomass, and not as explicit properties that may vary independently due to differences in microbial life strategies or microbe-substrate interactions.

We investigated how kinetic properties and temperature sensitivity of soil exoenzymes vary with soil depth, possibly representing depth-dependent traits associated with microbiomes adapted to distinct soil environments. Given the role of forests as globally critical C reservoirs (Griscom et al., 2017), we investigated exoenzymes in soils from a temperate coniferous forest site, which has been shown to have lost substantial subsoil C in response to experimental warming (Hicks Pries et al., 2017; Soong et al., 2021). The soil profile at this site is also known to reflect typical gradients in decreasing soil C and temperature range (Hicks Pries et al., 2017; Soong et al., 2021). We hypothesized that: (i) enzyme *V*_max_ declines with depth, in concert with declines in substrate concentrations and overall nutrient demand; (ii) enzyme affinities and catalytic efficiencies increase with depth to maximize resource acquisition under low substrate concentrations; (iii) variation of kinetic properties with depth differs between C-, N- and P-acquiring enzymes, reflecting differences in relative substrate availability and demand; (iv) temperature sensitivity of exoenzymes increases with depth, reflecting selection of enzymes adapted to lower and narrower temperature ranges in deeper soils. We determined the MM kinetics and catalytic efficiencies of the hydrolytic enzymes β-glucosidase (BG), leucine aminopeptidase (LAP) and acid phosphatase (AP) (involved in C, N and P acquisition, respectively), as a function of both soil dry mass and microbial biomass C, in soils collected at six depths down to 90 cm. Furthermore, we investigated enzyme temperature sensitivity based on the Arrhenius model and *Q*_10_ coefficients, and on the MMRT model over six temperatures between 4–50°C, following a fully factorial experimental design considering substrate type and concentration, soil depth and temperature.

## Materials and Methods

### Site Description and Sample Collection

Soil samples were collected at the University of California Blodgett Experimental Forest, Sierra Nevada, CA, United States (120°39′40″ W; 38°54′43″ N), described by Hicks Pries et al. (2018). Briefly, Blodgett forest is located in a Mediterranean climate with mean annual precipitation of 1,660 mm and a mean annual air temperature of 12.5°C. The soil was classified as Alfisol of granitic origin, and has a developed O horizon. The site is a mixed coniferous forest with ponderosa pine (*Pinus ponderosa*), sugar pine (*Pinus lambertiana*), incense cedar (*Calodefrus decurrens*), white fir (*Abies concolor*) and douglas fir (*Pseudotsuga menziesii*) as dominant tree species. The mean annual soil temperature ranges between 11.5 and 10.4°C at 5 and 100 cm depths, respectively, although soil temperatures vary annually between 0–29°C, 1–19°C and 2–16°C at 5, 30, and 100 cm depth, respectively. Three soil cores were collected in July 2019 using a 4.78 cm diameter soil corer with a 10 kg hand-held slide-hammer. The surface litter layer of the O horizon was removed prior to sampling, and mineral soil samples were recovered sequentially in 10 cm increments down to 90 cm depth. Samples were kept cold during transportation to the laboratory, where they were sieved to 2 mm and stored at 4°C. Samples were analyzed within approximately a week of collection. To ensure the accessibility and discoverability of the samples generated here, and to align with the National Science Foundation’s guidelines of effective data practices, all samples have been registered with IGSN Global Sample Numbers through the System for Earth Sample Registration (SESAR). SESAR is maintained by the GeoInformatics Research Group of the Lamont-Doherty Earth Observatory at https://www.geosamples.org/. Sample IGSNs are shown in Supplementary Table 1.

### Exoenzyme Activity Assays

Extracellular hydrolytic enzyme activities were determined fluorometrically according to standard assays (German et al., 2011b) at six depth intervals, following the experimental design in Table 1. Briefly, we used the methylumbelliferone (MUF)-linked substrates MUF-β-D-glucopyranoside and MUF-phosphate for determination of β-glucosidase (BG) and acid phosphatase (AP) activities, respectively. Leucine aminopeptidase (LAP) activity was determined using the substrate L-leucine-7-amido-4methylcoumarin (AMC). Assays were performed for each of six soil depths from each of three replicate soil cores, by combining 200 μL of soil homogenate with 50 μL of fluorogenic substrate solution in each microplate well. Soil homogenates were prepared with 1 g of fresh soil in 100 mL 50 mM acetate buffer with pH 5.5, by mixing with a regular blender. The same buffer was used to prepare all substrate solutions, soil homogenates, serial dilutions of standards in the absence or presence of soil homogenate (quenching controls), blank quenching controls without standards, and blank controls in the absence or presence of each of the eight substrate concentrations. Standards in the presence or absence of soil homogenates (quenched standards) were prepared over six 1:10 serial dilutions, from 0.625 to 20 μM for MUF, and 0.3125 to 10 μM for AMC. MUF and AMC standards without soil homogenates, blank controls with only substrates, and blank quenching controls with soil homogenates, but no MUF or AMC standards, were performed in duplicate. Each enzyme was assayed individually over a range of eight substrate concentrations, as follows: 10, 30, 60, 100, 150, 250, 450, and 800 μM for BG; 10, 20, 40, 70, 110, 190, 350, and 600 μM for LAP; and 10, 40, 80, 130, 200, 350, 700, and 1200 μM for AP (Table 1). Parallel assays for each sample, enzyme and substrate concentration were performed in black microplates individually covered with lids to avoid evaporation, and incubated in the dark at 4, 10, 16, 25, 35, or 50°C. Fluorescence was recorded (excitation: 365 nm, and emission: 450 nm) after approximately 1, 4, and 24 h to determine the optimal incubation time. Four analytical replicates were measured per sample for each combination of enzyme, substrate concentration and temperature. A set of standards, and blank, substrate and quenching controls was incubated together with each batch of assays at each temperature, to correct fluorescence measurements for temperature-specific effects in the assays. Incubation temperatures were selected in order to capture the unimodal response predicted by MMRT with *T*_opt_-values well above native temperatures, as observed by previous studies of exoenzymes from temperate environments (Alster et al., 2016b), while including the temperature range and approximate seasonal averages at our experimental site.

**TABLE 1:** Experimental set-up of enzyme potential activity assays.

Enzyme	Fluorogenic substrate	Substrate concentration (μM)	Soil depth (cm)	Temperature (°C)	Incubation time (h)
β-glucosidase (BG)	4-methylumbelliferyl-β-D-glucopyranoside	10, 30, 60, 100, 150, 250, 450, 800	0–10	4	2
EC 3.2.1.21			10–20	10	4

Leucine aminopeptidase (LAP)	L-leucine-7-amido-4-methylcoumarin	10, 20, 40, 70, 110, 190, 350, 600	30–40	16	24
EC 3.4.11.1			50–60	25	

Acid Phosphatase (AP)	4-methylumbelliferyl phosphate	10, 40, 80, 130, 200, 350, 700, 1200	60–70	35	
EC 3.1.3.2			80–90	50	

### Microbial Biomass C and Dissolved C and N Pools

Microbial biomass C (MBC), dissolved organic C (DOC) and total dissolved N (TDN) were determined at every 10 cm depth interval between 0–90 cm depth. MBC was estimated using the chloroform-fumigation extraction method (Brookes et al., 1985). Five-gram soil samples were fumigated in 50 mL closed vials containing a jumbo cotton ball soaked with ethanol-free chloroform over, but not touching, the soil, for 7 days, with chloroform replenished on day 4. Fumigated and a non-fumigated soil samples were extracted with 25 mL of 0.5 M K_2_SO_4_ on an orbital shaker table for 60 min, then gravity filtered through pre-leached #42 Whatman filter paper, and frozen until further analysis. DOC and TDN in fumigated and non-fumigated samples were quantified using a Lotix Combustion TOC/TN Analyzer (Teledyne Tekmar, Mason, OH, United States). No correction factor (k_EC_) was applied to account for incomplete microbial biomass lysis during the fumigation.

### Data Analyses

All data manipulations and analyses were performed in R versions 3.6.1-4.0.4 (R Core Team, 2020). All enzyme trait abbreviations and definitions are indicated in Table 2. Net fluorescence in the enzyme assays, including quenching corrections, were calculated following German et al. (2011b). Negative values due to analytical error were excluded from the dataset (0.8%); analytical outliers were further identified based on the Interquartile Range method, and a maximum of one value was excluded out of the four analytical replicates per sample. In total, these procedures excluded 1917 out of 26784 data-points (7.2%). Enzyme maximal velocity (*V*_max_) and Michaelis constants (*K*_m_) were computed by fitting a 2-parameter Michaelis-Menten (MM) model over all analytical replicates of each of eight substrate concentrations using the *drm* function in the *drc* package (Ritz et al., 2015), with a data-driven self-starter function specific to the model. Following preliminary analyses, and when necessary, we excluded data points corresponding to one of the eight individual substrate concentrations for which all analytical replicates consistently did not fit the distribution of the remaining data (i.e., due to inhibition at high concentrations or technical errors during assay preparation). In order to alleviate variance heterogeneity of analytical replicates between substrate concentrations, we applied a Box-Cox transformation to all models using the *boxcox* function in the *drc* package (Ritz et al., 2015). Comparison between the parameters *V*_max_ and *K*_m_ estimated based on transformed and non-transformed models showed that Box-Cox transformation improved the fit of models with substantial analytical variance, but had a marginal or no effect on parameters estimated by models with initial good fit. Individual models yielding nonsignificant *V*_max_ or *K*_m_ estimates (*p* > 0.05) after Box-Cox transformation were considered to have bad fit and were thus excluded from further analyses (excluded 11 out of 324 models). To determine the optimal assay incubation time at each temperature, we compared MM models fit to data collected after each of three sequential incubation periods (1, 4, and 24 h). We selected the minimum incubation period necessary to reach the highest *V*_max_-value, under the assumptions that lower *V*_max_-values reflected either insufficient incubation time for reactions to reach saturation, decrease in activity, or loss of fluorescence due to prolonged incubation after saturation had been reached. The same incubation period was consistently selected for each batch of assays performed at the same temperature. *V*_max_ was expressed per mass of dry soil as *V*_max/ds_ (nmol g^–1^ h^–1^) and per unit of microbial biomass C (MBC) as *V*_max/MBC_ (nmol μg MBC^–1^ h^–1^). The apparent catalytic efficiency (CE_ds_) was calculated as:


$$\frac{V_{\max/\text{ds}}}{K_{\text{m}}}$$

and the biomass-specific catalytic efficiency (CE_MBC_) as:


$$\frac{V_{\max/\text{MBC}}}{K_{\text{m}}}$$

*Q*_10_ coefficients were calculated over the full experimental temperature range (six temperatures from 4 to 50°C) and over a realistic field range (five temperatures from 4 to 35°C) following the approach by Allison et al. (2018). Briefly, the degree of change in *V*_max_, *K*_m_ or CE per °C was inferred based on linear regressions between the natural logarithm of each parameter and temperature, and converted to *Q*_10_-values based on the relationship:


$$Q_{10}=\exp\left(10\times slope\right)$$

The Arrhenius activation energy (*E*_a_) was calculated based on the slope of the linear regression between ln(*V*_max_) and 1/*T*, and the relationship:


$$-E_{a}=slope\times R$$

where *T* is the temperature in kelvin and *R* is the universal gas constant. Linear regression models were calculated using the *lm* function in the *stats* package native to R (R Core Team, 2020). The change in heat capacity (Δ*C*_p_^‡^), temperature optimum (*T*_opt_) and point of maximum temperature sensitivity (TS_max_) were calculated by fitting ln(*V*_max_) over the six incubation temperatures between 4–50°C using the Macromolecular Rate Theory (MMRT) model, according to the equations and definitions described by Alster et al. (2020). The reference temperature *T*_0_ was set to 315 K to best match the measured data, following the recommendations by Alster et al. (2020). Model fit comparisons were based on the Akaike Information Criterion (AIC) and respective relative likelihoods, corrected AIC (AICc), and Bayesian Information Criterion (BIC), following the guidelines by Burnham and Anderson (2004). AIC, BIC, and adjusted *R*^2^-values of the linear models were extracted from the linear regression model computed with the *lm* function in the *stats* package (R Core Team, 2020). AICc of all models, and AIC and BIC of the nonlinear models were calculated using the R package *AICcmodavg* (Mazerolle, 2020). One-way and two-way Analyses of Variance (ANOVA) were performed with the *aov* function, followed by *post hoc* Tukey’s tests using the function *TukeyHSD* with *p*-values adjusted for multiple comparisons, using the *stats* package (R Core Team, 2020). Compact letter displays for the Tukey’s tests were computed with the function *HSD.test* in the package *agricolae* (de Mendiburu and Yaseen, 2020). Assumptions of ANOVA were tested based on Levene’s tests with the *leveneTest* function in the package *car* (Fox and Weisberg, 2019), Shapiro-Wilk tests with the *shapiro.test* function in the package *stats* (R Core Team, 2020), skewness of residuals with the *skewness* function in the package *agricolae* (de Mendiburu and Yaseen, 2020), and plots of homogeneity of residuals’ variance and normality of residuals (Q-Q plots). Data was ln-transformed as necessary, and all tests reported as significant were based on a *p*-value < 0.05. Figure displays were prepared with the package *cowplot* (Wilke, 2020). The maximum percentage of variation (i.e., decline) in kinetic parameters with depth, per temperature, was calculated as the percentage of difference between the highest and lowest values within the upper and lower depth intervals mentioned in the text, for example:


$$\frac{\max\left(V_{\max}^{0-20cm}\right)-\min\left(V_{\max}^{60-90cm}\right)}{\max\left(V_{\max}^{0-20cm}\right)}\times 100\%$$

As the ANOVA showed that variation in kinetic parameters with depth was not dependent on temperature, the percentages of variation with depth are reported as the average decline among all temperatures, per enzyme and kinetic parameter. The inconsistently high mean *K*_m_-values only at 16°C was excluded from those calculations. All raw and processed data, as well as the code used to parse and analyze them are available as Supplementary Material (see “Supplementary_Materials_File_Descriptions.pdf”).

**TABLE 2:** Enzyme trait abbreviations and definitions used this study.

	Enzyme trait	Definition
Michaelis-Menten kinetics	*V*_max/ds_	Maximum velocity per mass dry soil: maximum reaction rate at substrate saturation, on a soil mass basis.
	*V*_max/MBC_	Biomass-specific maximum velocity: maximum reaction rate at substrate saturation, per unit microbial biomass C.
	*k*_cat_	Turnover number, or catalytic rate constant: maximum number of substrate molecules converted to product per catalytic center per unit time.
	*K*_m_	Michaelis, or half-saturation, constant: inversely proportional to enzyme affinity.
	CE_ds_	Apparent catalytic efficiency (CE): ratio between *V*_max/ds_ and *K*_m_.
	CE_MBC_	Biomass-specific catalytic efficiency (CE): ratio between *V*_max/MBC_ and *K*_m_.
**Temperature sensitivity:** Arrhenius	*Q*_10_	Temperature coefficient: factor by which a rate changes with each 10°C change in temperature.
	*E*_a_	Activation energy: minimum amount of energy required for a reaction to occur.
**Temperature sensitivity:** Macromolecular Rate Theory	*T*_opt_	Temperature optimum: temperature at which the reaction rate is highest.
	TS_max_	Point of maximum temperature sensitivity: temperature at which the increase in reaction rate is highest.
	Δ*C*_p_^‡^	Change in heat capacity between enzyme–substrate and enzyme–transition state complexes, which defines the shape of the rate temperature response.

## Results

### Exoenzyme Kinetics Vary With Soil Depth

We determined the MM kinetics of the enzymes acid phosphatase (AP), β-glucosidase (BG), and leucine aminopeptidase (LAP) in soils collected at six depth intervals from triplicate soil cores down to 90 cm (0–10, 10–20, 30–40, 50–60, 60–70, and 80–90 cm), at six temperatures between 4 and 50°C (4, 10, 16, 25, 35, or 50°C) (Table 1). The activity of all enzymes showed typical MM behavior. Enzyme kinetic traits analyzed here and their definitions are indicated in Table 2.

**FIGURE 1:**
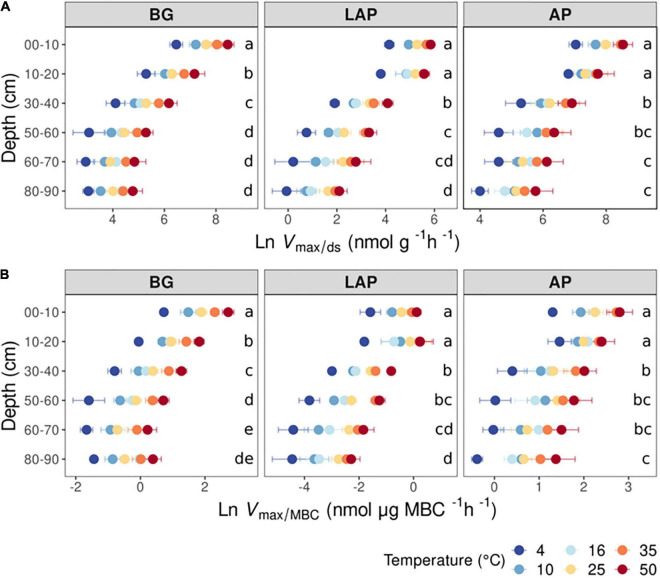
Enzyme maximum velocity (*V*_max_) at different depths and six temperatures from 4 to 50°C, expressed per **(A)** dry soil mass (*V*_max/ds_), or **(B)** microbial biomass C (*V*_max/MBC_). Two-way ANOVA with depth and temperature as interactive factors indicated that both depth and temperature had a significant effect on *V*_max_ of all enzymes (*p* < 0.05), but without interaction between the two factors (Table 3). Colors indicate incubation temperatures and letters indicate significant differences (*p* < 0.05) between depths per enzyme, based on Tukey’s tests after ANOVA tests. Error bars represent the standard error of the mean (*n* = 3).

The *V*_max_ of all three enzymes, estimated on a dry soil mass basis (*V*_max/ds_), declined significantly with soil depth over all temperatures (*p* < 0.001), and differences among depths were not dependent on temperature (i.e., no significant depth × temperature interaction) (Figure 1A and Table 3). Mean *V*_max/ds_ declined almost continuously from the soil surface (0–20 cm) down to 60–90 cm by up to 96.4 ± 0.4% (mean ± standard error; see Materials and Methods for details) across all enzymes and temperatures. This variation was only significant between three to four depth ranges, which differed between enzymes (Figure 1A, Supplementary Table 2): *V*_max/ds_ of BG declined progressively down to 60 cm, but not below that depth; *V*_max/ds_ of AP declined only over the mid-depth range, from 20 to 30 cm and from 40 to 60 cm; *V*_max/ds_ of LAP also did not vary within the upper 20 cm, but declined gradually down to a lower depth than that of AP, namely from 20 to 30 cm, from 40 to 50 cm and from 60 to 80 cm.

**TABLE 3:** Two-way fixed effects ANOVA of kinetic parameters with depth and temperature as independent factors, per enzyme.

		*V*_max/ds_			*V*_max/MBC_			*K*_m_			CE_ds_			CE_MBC_		
Enzyme	Factor	Df	*F-*value	*p-*value	Df	*F-*value	*p-*value	Df	*F-*value	*p-*value	Df	*F-*value	*p-*value	Df	*F-*value	*p-*value
BG	Depth	5	89.32	** < 0.05**	5	100.95	** < 0.05**	5	30.03	** < 0.05**	5	45.42	** < 0.05**	5	27.37	** < 0.05**
	Temperature	5	19.94	** < 0.05**	5	54.16	** < 0.05**	5	2.64	** < 0.05**	5	14.55	** < 0.05**	5	28.54	** < 0.05**
	Depth × Temperature	25	0.09	1.00	25	0.25	1.00	25	1.52	0.09	25	0.9	0.61	25	1.61	0.06
LAP	Depth	5	127.92	** < 0.05**	5	64.27	** < 0.05**	5	20.94	** < 0.05**	5	77.8	** < 0.05**	5	30.97	** < 0.05**
	Temperature	5	27.69	** < 0.05**	5	29.61	** < 0.05**	5	1.52	0.19	5	51.05	** < 0.05**	5	69.4	** < 0.05**
	Depth × Temperature	25	0.32	0.99	25	0.33	0.99	25	0.56	0.95	25	0.18	1.00	25	0.19	1.00
AP	Depth	5	44.43	** < 0.05**	5	29.08	** < 0.05**	5	25.22	** < 0.05**	5	3.2	** < 0.05**	5	1.47	0.212
	Temperature	5	9.72	** < 0.05**	5	20.23	** < 0.05**	5	1.53	0.19	5	7.61	** < 0.05**	5	14.31	** < 0.05**
	Depth × Temperature	25	0.15	1.00	25	0.26	1.00	25	0.32	0.99	25	0.09	1.00	25	0.15	1.00

*Differences were considered significant at p < 0.05 (values in bold font).*

Since the concentration of microbial biomass carbon (MBC) declined strongly with soil depth, especially over the upper 30 cm (Supplementary Figure 1), much of the decline in *V*_max/ds_ with depth may have been driven by lower microbial abundance. Therefore, we computed a biomass-specific *V*_max_, by expressing it per unit MBC (*V*_max/MBC_), under the assumption that cell lysis efficiency by chloroform fumigation was similar across samples, thus yielding comparable MBC estimates. *V*_max/MBC_ of all enzymes declined significantly down the soil profile over all temperatures, following the same trends as those of *V*_max/ds_ (*p* < 0.001) (Table 3, Supplementary Table 2, and Figure 1B). The overall decline in *V*_max/MBC_ between 10–20 and 60–90 cm was only 8% lower (88.4 ± 1.7%) than that of *V*_max/ds_, indicating that variation in *V*_max/ds_ did not depend primarily on microbial biomass concentration. Like *V*_max/ds_, *V*_max/MBC_ did not show a significant interaction between depth and temperature. Also similar to *V*_max/ds_, *V*_max/MBC_ of AP and LAP did not vary within the upper 20 cm and declined mostly from 20 to 30 cm (Figure 1B). *V*_max/MBC_ did not decline significantly over the mid-depth range for either AP or LAP, but it was significantly lower at 80–90 cm than at 30–40 cm for AP, and lower between 60 and 90 cm than at 30–40 cm for LAP. *V*_max/MBC_ of BG declined more consistently down to 70 cm over all temperatures, but did not vary further.

**FIGURE 2:**
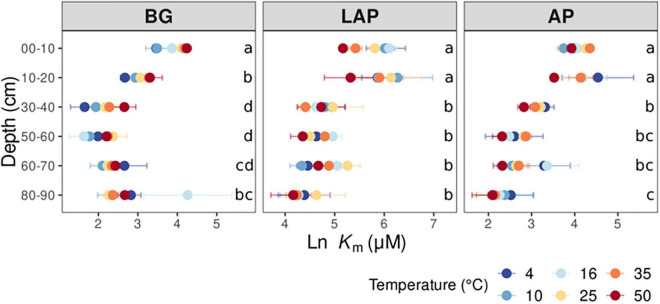
Enzyme Michaelis constant (*K*_m_) at different depths and six temperatures from 4 to 50°C. Two-way ANOVA with depth and temperature as interactive factors indicated that depth had a significant effect on *K*_m_ of all enzymes (*p* < 0.05), whereas temperature only had a significant effect on *K*_m_ of BG (Table 3). There was no depth × temperature effect. Colors indicate incubation temperatures and letters indicate significant differences (*p* < 0.05) between depths per enzyme, based on Tukey’s tests after ANOVA tests. Error bars represent the standard error of the mean (*n* = 3).

*K*_m_ also declined (i.e., enzyme affinity increased) significantly with depth for all enzymes across temperatures (*p* < 0.001) with no significant interaction between depth and temperature (Table 3, Supplementary Table 2). However, *K*_m_ declined less with depth than *V*_max/ds_ or *V*_max/MBC_, and mainly between the upper 20 cm and lower depths, by up to 85.6 ± 1.3%, with some differences between enzymes. *K*_m_ of AP and BG declined with depth following trends similar to those of their *V*_max_ (Figure 2): *K*_m_ of AP declined mainly from 20 to 30 cm and remained relatively constant down to 80 cm, although it was significantly lower at 80–90 cm than at 30–40 cm; *K*_m_ of BG declined consistently down to 40 cm, without further variation (despite a spuriously high mean *K*_m_ at 80–90 cm only at 16°C). Unlike its *V*_max_, the *K*_m_ of LAP only declined from 20 to 30 cm, and did not vary significantly below that depth.

**FIGURE 3:**
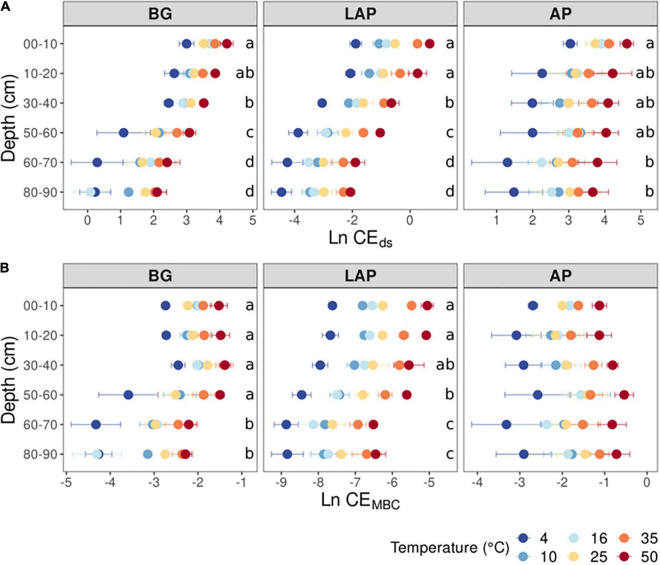
Enzyme catalytic efficiency at different depths and six temperatures from 4 to 50°C, calculated on a **(A)** dry soil mass basis (CE_ds_), or a **(B)** microbial biomass C basis (CE_MBC_). Two-way ANOVA with depth and temperature as interactive factors indicated that both depth and temperature had a significant effect on CE_ds_ and CE_MBC_ of all enzymes (*p* < 0.05), except for no effect of depth on CE_MBC_ of AP (Table 3). There was no depth × temperature effect. Colors indicate incubation temperatures and letters indicate significant differences (*p* < 0.05) between depths per enzyme, based on Tukey’s tests after ANOVA tests. Error bars represent the standard error of the mean (*n* = 3).

The apparent (i.e., observed) catalytic efficiency (CE_ds_), estimated as the ratio between *V*_max/ds_ and *K*_m_, reflects the catalytic efficiency of the enzyme pool present per mass of soil, regardless of microbial abundance or enzyme demand. CE_ds_ declined significantly across temperatures for all enzymes (*p* < 0.001), also without a significant interaction between depth and temperature (Table 3, Supplementary Table 2). The CE_ds_ of BG and LAP followed similar trends and declined mainly over the mid-depth range by up to 90.5 ± 1.1% over all temperatures, without significant variation in the upper 20 cm, or below 60 cm (Figure 3A). CE_ds_ of AP declined much less through the soil profile (58.1 ± 3.0%), and it was only significantly lower at 60–90 cm than in the upper 10 cm. Biomass-specific catalytic efficiency (CE_MBC_), estimated as the ratio between *V*_max/MBC_ and *K*_m_, represents the inherent catalytic efficiency of the enzyme pool produced by the local microbiome, as a function of its specific enzyme production capacity and demand. CE_MBC_ declined significantly with depth for BG and LAP (*p* < 0.001) across temperatures, but not for AP (Table 3, Supplementary Table 2, and Figure 3B). CE_MBC_ of BG and LAP varied less with depth than other kinetic properties, and declined significant mainly below 60 cm by up to 71.8 ± 2.5% (Figure 3B). These declines in CE_MBC_ reflected the decline in *V*_max/MBC_ at lower depths, where *K*_m_ remained relatively constant. Like for all other kinetic parameters, depth-dependent differences in CE_MBC_ were not dependent on temperature (Table 3).

As there was no significant interaction between depth and temperature for any kinetic parameter (Table 3), we performed a random effects ANOVA with depth as independent variable and temperature as block variable, to control for possible confounding effects of the latter on depth-dependent differences. This analysis yielded the same results as the fixed effects ANOVA reported above, with the single exception that *V*_max/ds_ of AP declined significantly also between 50–60 cm and 80–90 cm (data not shown).

### Exoenzyme Kinetics Vary Among Enzymes as a Function of Soil Depth

All kinetic properties varied significantly among enzymes at all depths and across temperatures (*p* < 0.001), but there was only a significant interaction between enzyme and temperature in the upper 10 cm for *K*_m_, CE_ds_ and CE_MBC_ (*p* < 0.001) (Supplementary Table 3). *V*_max_ differed significantly among all enzymes at all depths, with AP having consistently the highest values, followed by BG and then LAP (*p* < 0.05) (Supplementary Table 4 and Figures 1A,B). *K*_m_ differed significantly among all enzymes between 10 and 60 cm (*p* < 0.05), but it did not differ between AP and BG in the upper 10 cm or below 60 cm (Supplementary Table 4 and Figure 2). LAP had always the highest *K*_m_ (i.e., lowest affinity). In contrast, BG had always the lowest *K*_m_, at least at depths where it was significantly different than that of AP (i.e., between 10 and 60 cm). CE_MBC_ differed significantly among all enzymes in the upper 10 cm and below 50 cm (*p* < 0.05), but not between AP and BG from 10 to 40 cm (Supplementary Table 4 and Figure 3B). Similar to *V*_max_, the CE_MBC_ of LAP was always the lowest among enzymes, followed by those of BG and then AP at depths where it differed significantly (Supplementary Table 4 and Figure 3B). As CE_MBC_ was calculated based on the same MBC value for all enzymes at each depth, CE_ds_ varied between enzymes similarly to CE_MBC_ (Supplementary Table 4 and Figure 3A).

**FIGURE 4:**
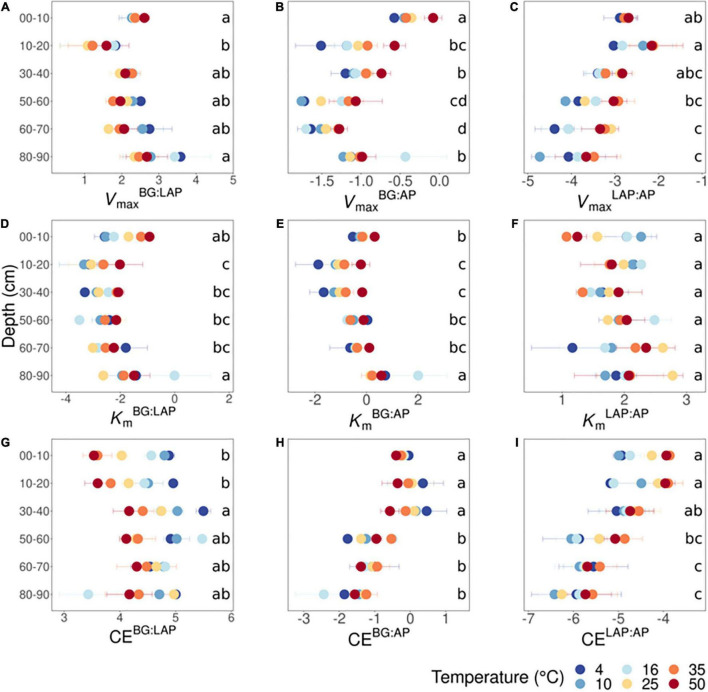
Ratios between kinetic parameters of BG, LAP and AP at different depths and six temperatures from 4 to 50°C. **(A)** *V*_max_^BG:LAP^, **(B)** *V*_max_^BG:AP^, **(C)** *V*_max_^LAP:AP^, **(D)** *K*_m_^BG:LAP^, **(E)** *K*_m_^BG:AP^, **(F)** *K*_m_^LAP:AP^, **(G)** CE^BG:LAP^, **(H)** CE^BG:AP^, and **(I)** CE^LAP:AP^. Two-way ANOVA with depth and temperature as interactive factors indicated that depth had a significant effect on all ratios (*p* < 0.05), except on *K*_m_^LAP:AP^, whereas temperature had a significant effect only on *V*_max_^BG:AP^, *V*_max_^LAP:AP^ and CE^BG:LAP^ (Supplementary Table 5). There was a depth × temperature effect only on CE^BG:LAP^. Colors indicate incubation temperatures and letters indicate significant differences (*p* < 0.05) between depths per ratio, based on Tukey’s tests after ANOVA tests. Error bars represent the standard error of the mean (*n* = 3); dots without error bars represent data-points with *n* < 3 (data excluded due to Michaelis-Menten models with poor fit).

We investigated the ratios between kinetic properties (*V*_max_, *K*_m_, and CE) of the three different enzymes as indicators for variation in relative nutrient demand through the soil profile. Based on two-way fixed effects ANOVA with depth and temperature as independent factors, all kinetic ratios between enzymes varied significantly with depth over all temperatures, with the exception of ratios between *K*_m_ of LAP and AP (*K*_m_^LAP:AP^) (*p* < 0.005) (Supplementary Table 5). The effects of temperature on kinetic ratios are described in a separate section below. *V*_max_^BG:LAP^ declined significantly from 10 to 20 cm, followed by a suggestive continuous increase down to 90 cm, although it was only significantly higher at 80–90 cm than at 10–20 cm (Figure 4A). *V*_max_^BG:AP^ declined with depth down to 70 cm, mainly from 10 to 20 cm and from 40 to 60 cm, followed by a significant increase from 70 to 90 cm that appeared to result partially from a spurious high value only at 16°C, among all six temperatures (Figure 4B). *V*_max_^LAP:AP^ generally declined with depth below 20 cm, but this variation was mainly significant between the upper 20 cm and the lower 30 cm (i.e., from 60 to 90 cm) (Figure 4C). *K*_m_^BG:LAP^ and *K*_m_^BG:AP^ followed trends similar to those of their corresponding *V*_max_ ratios: *K*_m_^BG:LAP^ declined significantly from 10 to 20 cm and remained relatively invariant through the profile (Figure 4D), whereas *K*_m_^BG:AP^ declined from 10 to 20 cm, followed by a suggestive but non-significant increase down to 70 cm (Figure 4E). The significant increases in *K*_m_^BG:LAP^ and *K*_m_^BG:AP^ at 80–90 cm were likely driven, at least partially, by the same spurious high *K*_m_-value of BG only at 16°C mentioned above. In contrast, *K*_m_^LAP:AP^ did not vary significantly with depth nor show any apparent trends (Figure 4F). CE^BG:LAP^ increased significantly from the upper 20 cm to a depth of 30–40 cm, although below that depth it did not vary significantly from any upper depths (Figure 4G). CE^BG:AP^ did not vary within the upper 40 cm, but it was significantly lower below 50 cm (Figure 4H). CE^LAP:AP^ declined with depth below 20 cm, but this variation was only significant between the upper 40 cm and the lower 30 cm (from 60 to 90 cm) (Figure 4I). This variation in CE^LAP:AP^ with depth mirrored the general trend of *V*_max_^LAP:AP^, as *K*_m_^LAP:AP^ was relatively invariant though the soil profile.

### Temperature Sensitivity of Exoenzymes Is Similar Through the Soil Profile

**FIGURE 5:**
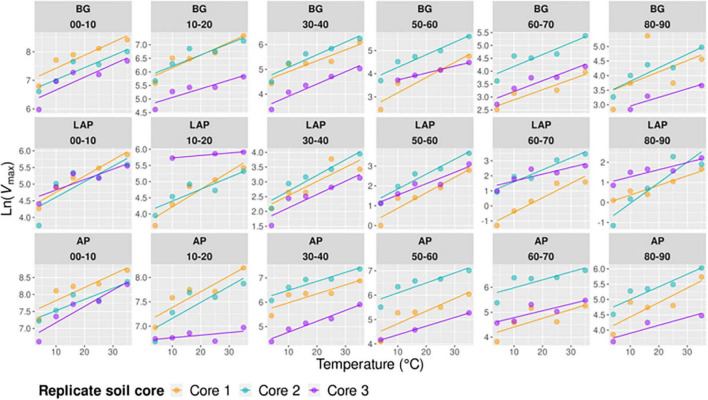
Arrhenius models of *V*_max_ over five temperatures from 4 to 35°C per enzyme, depth, and replicate core sample.

**FIGURE 6:**
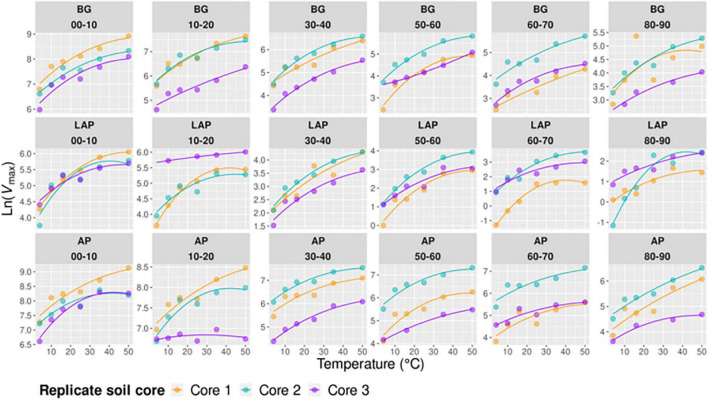
Macromolecular Rate Theory (MMRT) models of *V*_max_ over six temperatures from 4 to 50°C per enzyme, depth, and replicate core sample.

Temperature had an overall significant effect on *V*_max_, CE_MBC_ and CE_ds_ of all enzymes, and on *K*_m_ of only BG, over the whole soil profile (*p* < 0.001) (Table 3 and Figures 1–3). The temperature sensitivity of enzyme *V*_max_ (*V*_max/MBC_ and *V*_max/ds_ scale identically with temperature at each depth) was further determined using a linear Arrhenius model and *Q*_10_ coefficient over the full temperature range tested (4–50°C, *n* = 6) (Supplementary Figure 2) and a realistic *in situ* soil range (4–35°C, *n* = 5) (Figure 5), as well as based on the temperature optimum (*T*_opt_), point of maximum temperature sensitivity (TS_max_) and change in heat capacity (Δ*C*_p_^‡^) estimated using the non-linear MMRT model over the full temperature range (4–50°C, *n* = 6) (Figure 6). Enzyme thermal traits analyzed here and their definitions are indicated in Table 2. Although the fits of Arrhenius and MMRT models did not differ substantially, overall comparisons consistently favored MMRT, suggesting that it provided a more realistic representation of enzyme temperature response (see extended Results and Discussion in Supplementary Information).

**FIGURE 7:**
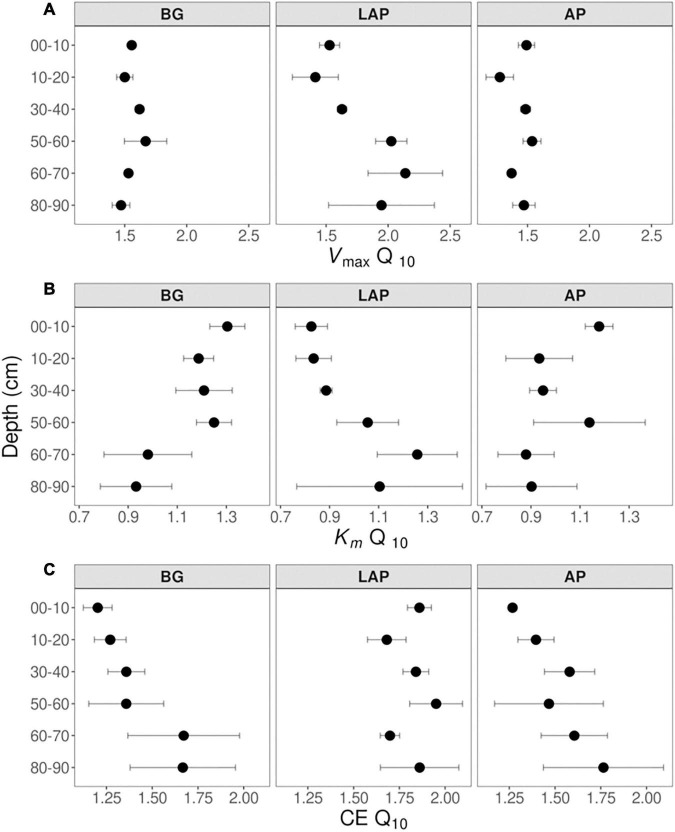
*Q*_10_ of Michaelis-Menten kinetic parameters over five temperatures from 4 to 35°C at different depths. **(A)** *V*_max_, **(B)** *K*_m_, and **(C)** CE. Differences between depths are not significant (*p* > 0.05), based on one-way ANOVA tests per enzyme (Table 4). Error bars represent the standard error of the mean (*n* = 3).

Despite some suggestive, depth-dependent trends in the *Q*_10_-values of *V*_max_, *K*_m_, and CE, they did not vary significantly with depth for any enzyme (Table 4), whether estimated over a realistic *in situ* soil temperature range (4–35°C) (Figure 7), or the full experimental temperature range (4–50°C) (Supplementary Figure 3). Both temperature ranges yielded similar *Q*_10_-values that varied within a narrow range, although *Q*_10_-values over 4–50°C were slightly lower than those over 4–35°C due to a frequent decline in the response rate of *V*_max_ between 35 and 50°C (Supplementary Table 8). Therefore, only *Q*_10_-values over the realistic *in situ* soil temperature range are henceforth presented. The *Q*_10_ of *V*_max_ was consistently above 1, indicating a positive effect of temperature on *V*_max_ across depths of 1.44 ± 0.03, 1.56 ± 0.03, and 1.78 ± 0.10 (mean ± se) for AP, BG and LAP, respectively (Figure 7A and Supplementary Table 8). Mean activation energies (*E*_a_) estimated from the same linear relationships were 25.69 ± 1.74, 31.50 ± 1.45, and 39.33 ± 3.98 kJ mol^–1^ K^–1^ (mean ± se) across depths for AP, BG, and LAP respectively (Supplementary Table 8). The *Q*_10_ of *K*_m_ varied between 0.83 and 1.30 across enzymes and depths, although *K*_m_ was, on average, relatively insensitive to temperature compared to other parameters, with mean *Q*_10_-values across depths of 1.00 ± 0.06, 1.14 ± 0.05, and 0.99 ± 0.07 (mean ± se) for AP, BG and LAP, respectively (Figure 7B and Supplementary Table 8). This was consistent with the two-way ANOVA showing that temperature had generally no significant effect on *K*_m_. The significant effect of temperature on the *K*_m_ of BG detected by the two-way ANOVA was likely due to the spurious high *K*_m_ of BG only at 16°C at 80–90 cm (Table 3 and Figure 2), which was not reflected on the *Q*_10_ computed across temperatures. The *Q*_10_ of CE was consistently above 1, similar to that of *V*_max_, with similar overall mean values across enzymes: 1.51 ± 0.08, 1.42 ± 0.08, and 1.82 ± 0.05 (mean ± se) for AP, BG, and LAP respectively (Figure 7C and Supplementary Table 8).

**TABLE 4:** One-way ANOVA of temperature sensitivity estimates with depth per enzyme.

	*V*_max_ *Q*_10_			*K*_m_ *Q*_10_			CE *Q*_10_			*T*_opt_			TS_max_			Δ*C*_p_^‡^		
Enzyme	Df	*F*-value	*p*-value	Df	*F*-value	*p*-value	Df	*F*-value	*p*-value	Df	*F*-value	*p*-value	Df	*F*-value	*p*-value	Df	*F*-value	*p*-value
BG	5	0.81	0.56	5	1.71	0.21	5	1.00	0.46	5	0.66	0.67	5	0.72	0.62	5	0.84	0.56
LAP	5	1.59	0.24	5	1.08	0.42	5	0.73	0.62	5	0.80	0.57	5	3.63	**<0.05**	5	0.80	0.57
AP	5	1.71	0.21	5	0.79	0.58	5	0.71	0.63	5	1.98	0.16	5	1.92	0.17	5	1.25	0.35

*Q_10_-values were calculated between 4–35°C, and the MMRT model parameters T_opt_, TS_max_, and ΔC_p_^‡^ between 4–50°C. Differences were considered significant at p < 0.05 (values in bold font).*

**FIGURE 8:**
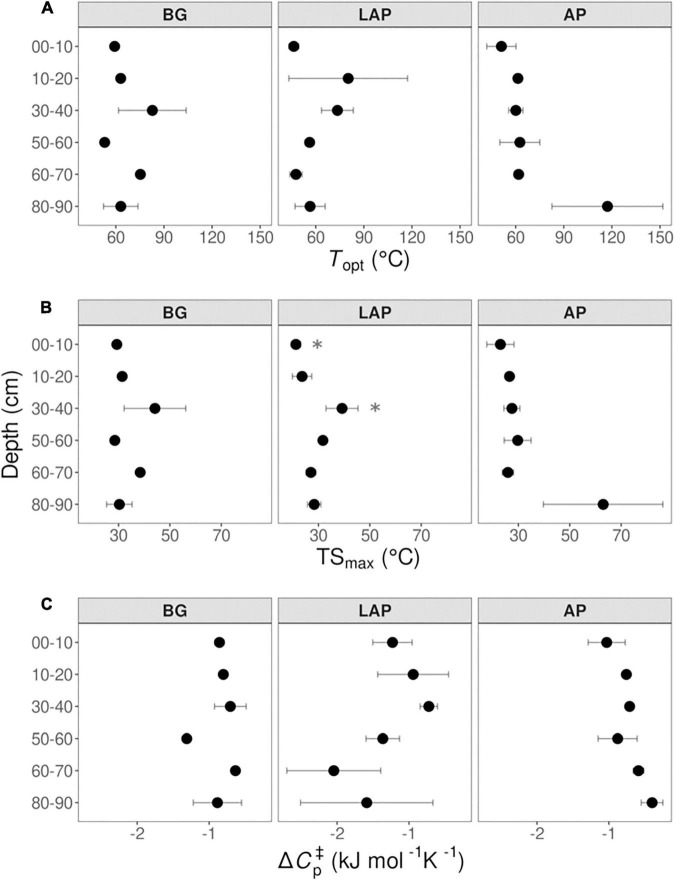
Macromolecular Rate Theory (MMRT) model estimates over six temperatures from 4 to 50°C at different depths. **(A)** *T*_opt_, **(B)** TS_max_, and **(C)** Δ*C*_p_^‡^. Differences between depths are not significant (*p* > 0.05), based on one-way ANOVA tests per enzyme (Table 4), except for TS_max_ of LAP between the depths indicated by asterisks (*). Error bars represent the standard error of the mean (*n* = 3).

None of the temperature sensitivity parameters estimated by MMRT –*T*_opt_, TS_max_ and Δ*C*_p_^‡^– varied significantly with depth (with one exception; see below) (Figure 8 and Table 4). These parameter estimates showed considerable variability among replicates, and estimates from models with poor fit to MMRT’s predicted behavior (*T*_opt_ or TS_max_ < 0°C, or > 200°C; four out of 54 total models) were excluded from the analysis, likely reducing the statistical power of few pairwise comparisons between depths and enzymes. Mean *T*_opt_ and TS_max_ were consistent across depths and enzymes, with mean values of 65.19 ± 3.74°C and 31.63 ± 1.98°C (mean ± se), respectively (Figures 8A,B and Supplementary Table 8). TS_max_ of LAP was significantly different between the 0–10 and 30–40 cm depth intervals (*p* < 0.05), which was the only exception to otherwise non-significantly different parameter estimates across either enzymes or depths. Mean Δ*C*_p_^‡^-values were similar between AP and BG across depths, with a combined mean value of −0.79 ± 0.06 kJ mol^–1^ K^–1^ (mean ± se) (Figure 8C and Supplementary Table 8). Δ*C*_p_^‡^ of LAP spanned a broader range of values (−1.32 ± 0.21, mean ± se), mainly due to suggestive, albeit non-significant, lower values at depths below 60 cm.

### Temperature Sensitivity of Some Kinetic Properties Varies Between Exoenzymes but Only at Discrete Depths

Temperature had a significant, positive effect on the *V*_max_ ratio of BG to AP (*V*_max_^BG:AP^), and LAP to AP (*V*_max_^LAP:AP^) across depths, and a significant, negative effect on ratios between catalytic efficiencies of BG and LAP (CE^BG:LAP^) (*p* < 0.05) (Supplementary Table 5 and Figures 4A–I). This indicated that *V*_max_ and CE of those enzymes were differently affected by temperature. CE^BG:LAP^ was also subject to a depth × temperature interaction (*p* < 0.05), suggesting that the different effects of temperature on CE of BG and LAP were depth-dependent. The *Q*_10_ of *V*_max_ and CE, and MMRT’s Δ*C*_p_^‡^ varied significantly between enzymes across the soil profile (*p* < 0.05), but there was no enzyme × depth interaction in the two-way ANOVA (Table 5). One-way ANOVA between enzymes at each depth interval showed that *Q*_10_-values varied significantly only between some enzymes and at discrete depths (Supplementary Tables 9–10). While the *Q*_10_ of *V*_max_ was only significantly different between AP and LAP at 60–70 cm (*p* < 0.05), this analysis also showed that the *Q*_10_ of *K*_m_ was significantly lower for LAP in the upper 10 cm relative to the other two enzymes (*p* < 0.05). This difference suggested that the affinity of LAP might increase with temperature (i.e., lower *K*_m_) at this depth (*Q*_10_ = 0.83 ± 0.07, mean ± se), compared to those of AP (*Q*_10_ = 1.18 ± 0.06) or BG (*Q*_10_ = 1.30 ± 0.07). The *Q*_10_ of CE was significantly higher for LAP in the upper 10 cm (1.86 ± 0.07, mean ± se) relative to those of AP (1.27 ± 0.02) or BG (1.20 ± 0.08) (*p* < 0.05), reflecting the apparent negative effect of higher temperatures on the *K*_m_ of LAP at that depth. Although overall Δ*C*_p_^‡^-values varied significantly between enzymes across depths (i.e., two-way ANOVA) (Table 5), one-way ANOVA did not detect significant differences at any specific depth for any MMRT parameter (Supplementary Table 9).

**TABLE 5:** Two-way fixed effects ANOVA of temperature sensitivity estimates with enzyme type and depth as independent factors.

	*V*_max_ *Q*_10_			*K*_m_ *Q*_10_			CE *Q*_10_			*T*_opt_			TS_max_			Δ*C*_p_^‡^		
Factor	Df	*F*-value	*p*-value	Df	*F*-value	*p*-value	Df	*F*-value	*p*-value	Df	*F*-value	*p*-value	Df	*F*-value	*p*-value	Df	*F*-value	*p*-value
Enzyme	2	7.95	**<0.05**	2	2.14	0.13	2	7.77	**<0.05**	2	0.81	0.45	2	0.84	0.44	2	4.33	**<0.05**
Depth	5	2.42	0.05	5	0.66	0.66	5	1.39	0.25	5	1.15	0.35	5	1.74	0.15	5	0.67	0.65
Enzyme x Depth	10	1.24	0.30	10	1.35	0.24	10	0.55	0.84	10	0.88	0.56	10	0.99	0.47	10	0.85	0.59

*Q_10_-values were calculated between 4–35°C, and the MMRT model parameters T_opt_, TS_max_, and ΔC_p_^‡^ between 4–50°C. Differences were considered significant at p < 0.05 (values in bold font).*

## Discussion

We show that kinetic properties of the enzymes BG, LAP, and AP varied markedly through the soil profile at a temperate forest, even when accounting for the large variation in microbial biomass. Moreover, this variation in enzyme kinetics was independent from temperature, as kinetic properties varied similarly between soil depths over temperatures between 4 and 50°C. We also show that the temperature sensitivity of each enzyme is similar through the soil profile, based on both linear Arrhenius and non-linear MMRT models, although temperature can directly affect the relative kinetics between enzyme types at discrete depths. To our knowledge, this is the first study to investigate the MM kinetic properties of soil enzymes and their direct temperature sensitivity through the soil profile, in this case to 90 cm.

### Higher Exoenzyme Affinity, but Lower *V*_max_ and Catalytic Efficiency, Indicate Adaptation to Lower Substrate Availability and Distinct Microbial Life Strategies in Deeper Soils

As hypothesized, *V*_max/ds_ and *K*_m_ declined strongly with soil depth, but followed distinct trends that depended on enzyme type. The decline in *V*_max/ds_ of 96.4 ± 0.4% across enzymes and temperatures down to 90 cm indicated a drastic decline in enzyme production capacity, product demand, or substrate availability. This was consistent with the lower microbial biomass concentrations in deeper soils observed here, as generally reported across studies (Blume et al., 2002; Fierer et al., 2003a; Schnecker et al., 2014; Loeppmann et al., 2016a; Jones et al., 2018). Higher density of plant roots in surface soils may have also contributed to higher near-surface *V*_max/ds_, as shown particularly for BG in rooted soils when compared to fallow soils (Loeppmann et al., 2016b), and in rhizosphere hotspots when compared to bulk soil (Tian et al., 2020). Plants may induce higher enzyme *V*_max_ by promoting microbial growth through C-rich exudates, competing with microbes for N and P, or directly stimulating microbial enzyme production to enhance availability of assimilable products (Burns et al., 2013). Declines in activity of hydrolytic enzymes have been consistently observed down to depths between 50 and 420 cm in diverse soils, including from temperate, taiga and tropical forests, arctic tundra, grasslands and croplands (Taylor et al., 2002; Venkatesan and Senthurpandian, 2006; Gelsomino and Azzellino, 2011; Kramer et al., 2013; Schnecker et al., 2014, 2015; Stone et al., 2014; Loeppmann et al., 2016a; Jing et al., 2017; Darby et al., 2020; Dove et al., 2020). However, among the five studies from which we could retrieve at least approximate *V*_max/ds_-values, only Loeppmann et al. (2016a) observed mean declines in *V*_max/ds_ of BG, LAP and AP down to 70 cm similar to those observed here down to 90 cm (91.0 ± 2.2%), whereas others observed substantially smaller mean declines of approximately 67.5 ± 6.3% for AP and BG at depths between 55 and 110 cm (Venkatesan and Senthurpandian, 2006; Gelsomino and Azzellino, 2011; Stone et al., 2014). Consistent with this, only Loeppmann et al. (2016a) determined *V*_max_ based on a MM model over a series of enzyme substrate concentrations, as needed to estimate MM kinetics accurately. Potential enzyme activities have been frequently shown to correlate positively with MBC concentration (Perucci, 1992; Gelsomino and Azzellino, 2011; Stone et al., 2014; Ren et al., 2018), as *V*_max_ is linearly dependent on enzyme concentration, which in turn is largely dependent on microbial abundance. However, this is not always the case (Waring et al., 2014), as exoenzyme production may also be induced or repressed depending on substrate availability and product demand, following the evolutionary-economic mechanisms that regulate allocation of cellular resources (Allison et al., 2011; German et al., 2011a). Moreover, enzyme production varies between organisms and is subject to variable levels of regulation (Allison et al., 2011; Sinsabaugh and Shah, 2012; Burns et al., 2013). For example, some isozymes and enzyme types may be expressed at stable constitutional levels under specific conditions, as previously suggested for deep soils (Stone et al., 2014), whereas expression of others may be more strictly induced by cellular demands or environmental cues. Therefore, a biomass-specific *V*_max_ (*V*_max/MBC_) can be interpreted as a catalytic rate constant independent of microbial abundance, which represents the collective effect of inherent enzyme properties, and specific enzyme production and demand of the microbiome. Surprisingly, *V*_max/MBC_ of all enzymes declined nearly as much with depth as *V*_max/ds_, indicating that microbial abundance was not the primary driver of variation in *V*_max_. Loeppmann et al. (2016a) found similar trends only for BG and LAP, and only below 30–40 cm, as *V*_max/MBC_ increased from the surface to that depth and only then declined consistently down to 70 cm. These differences might have resulted from a steeper decline in substrate availability and microbial biomass in our soils, which are covered by a thick litter layer and have a shallow rhizosphere, compared to a more extensive rhizosphere (i.e., maize) and less surface litter in the soils studied by Loeppmann et al. (2016a). The consistent decline in *V*_max/MBC_ in deeper soils observed by us, and to some extent also Loeppmann et al. (2016a), contrasts with most other studies where *V*_max/MBC_ of AP, BG and LAP either increased, or did not vary with depth (Gelsomino and Azzellino, 2011; Kramer et al., 2013; Stone et al., 2014; Schnecker et al., 2015; Dove et al., 2020). We could only identify one study that detected a decline in *V*_max/MBC_ of BG (Taylor et al., 2002), although declines with depth have been more frequently observed for other enzymes (Taylor et al., 2002; Gelsomino and Azzellino, 2011; Schnecker et al., 2015). However, *V*_max_ in those studies was inferred from a single concentration of substrate across depths that was often below the saturation point necessary to reach the *V*_max_-values we observed, particularly in surface soils. We suggest that the apparent increase, or lack of variation, in *V*_max/MBC_ previously observed may have resulted from underestimating *V*_max_ in surface soils. Our results indicate that exoenzyme pools in deeper soils have inherently low potential reaction rates due to lower expression levels, lower substrate turnover, and/or catalytic efficiency than those in surface soils, possibly reflecting differences in microbial life strategies and substrate preferences.

The consistent increase in affinity (i.e., decrease in *K*_m_) of all enzymes with depth by 85.6 ± 1.3%, mainly between 20 and 60 cm, indicated a major decline in substrate availability at mid-depths consistent with the decline in DOC, TDN (Supplementary Figures 4A,B) and total C and N in these soils (Hicks Pries et al., 2017, 2018). This supported our hypothesis that persistently low substrate concentrations in deep soils select for microbes encoding isozymes with lower *K*_m_ in order to maintain relatively constant maximal catalytic rates (Sinsabaugh et al., 2014). *K*_m_-values largely above physiologic substrate concentrations would render enzyme activity entirely dependent on substrate availability, which, under deep soil conditions, would lead to suboptimal rates and provide limited return to the investment on enzymes. Conversely, higher substrate availability in surface soils through plant litter inputs likely alleviates the selective pressure on enzymes with high affinity. Inputs of readily assimilable compounds through root exudation may further alleviate this pressure by reducing the relative importance of continuously maintaining maximal depolymerization rates (Allison et al., 2011), similar to what has been observed for substrate induced respiration (Blagodatskaya et al., 2009). The variation in enzyme affinities with depth observed here indeed appeared to reflect the overall decline in root density –both fine and coarse roots– below 40 cm at this site (Hicks Pries et al., 2018). The *K*_m_ of BG particularly mirrored the continuous steep decline in fine root density down to 40 cm (Hicks Pries et al., 2018), whereas those of AP and LAP did not vary within the upper 20 cm. This suggests that availability of easily metabolizable C-containing compounds exuded by fine roots may have a particular regulatory effect on depolymerization of cellulose (Allison and Vitousek, 2005; Allison et al., 2011) through selection of microbes encoding BG isozymes with distinct affinities. This hypothesis is further supported by the higher affinities of cellulose-degradation enzymes, including BG, observed in fallow soils relative to rooted soils (Loeppmann et al., 2016b), and in bulk soils relative to rhizosphere hotspots (Tian et al., 2020). Moreover, microbes producing BG in the rhizosphere have been shown to be distinct from those in the detritusphere (Nuccio et al., 2020). It should be noted that, like in all other studies of enzyme kinetics or activity in environmental samples based on these assays, both *V*_max_ and *K*_m_ estimates may be affected by the native substrate concentrations (Sinsabaugh et al., 2014). At the same time, it seems implausible that such effects might have contributed substantially to the extreme declines in *V*_max_ and *K*_m_ with depth observed, given their interdependency and the necessary requirement for low *K*_m_ under the much lower substrate concentrations in deeper soils.

The catalytic efficiency (CE), also referred to as specificity constant (Gelsomino and Azzellino, 2011), can be determined as *k*_cat_/*K*_m_ when *K*_m_ exceeds the concentration of substrate present, which is typically the case under physiological conditions (Berg et al., 2002; Koshland, 2002). Therefore, CE not only represents a fundamental functional property under direct selective pressure, but also evolutionary tradeoffs between *k*_cat_ and affinity, which are themselves subject to selection (Sinsabaugh et al., 2014). However, *k*_cat_ expresses the maximum amount of substrate converted per unit of time, per enzyme unit (assuming a single catalytic center per enzyme), and thus it cannot be directly inferred from enzyme assays in complex environmental samples, such as soils, where specific enzyme concentrations are generally unknown and hard to quantify. In these cases, an apparent CE (CE_ds_) has been estimated as *V*_max/ds_/*K*_m_ (Moscatelli et al., 2012; Kujur and Kumar Patel, 2014; Triebwasser-Freese et al., 2015; Loeppmann et al., 2016a,b; Razavi et al., 2016), which represents the observed CE of the enzyme pool present per mass of soil, regardless of the specific production capacity and demand of the microbiome. In our soils, CE_ds_ of all enzymes declined significantly with depth, with CE_ds_ of BG and LAP declining consistently over the mid-depth (20 to 60 cm) by up to ∼90%, whereas that of AP varied much less. These results are consistent with those from the only other study that, to our knowledge, determined CE_ds_ in deep soils, where CE_ds_ of these same enzymes declined by 2- to 20-fold between the upper 40 cm and depths down to 70 cm (Loeppmann et al., 2016a). Tian et al. (2020) have also shown that CE_ds_ of BG and AP was higher in fertile soils than in nutrient-poor soils, consistent with higher CE_ds_ in surface soils with greater nutrient availability than deep soils. However, contrary to our initial expectations, CE based on biomass-specific *V*_max_ (CE_MBC_) either declined by up to ∼70% (BG and LAP) or did not vary (AP) with depth. We initially hypothesized that CE would increase with depth to maximize return on the investment in enzymes, given the scarce substrates provided by lower plant litter inputs and lower compensation by root exudates. Microbial communities adapted to these conditions would be expected to encode isozymes with higher affinity (i.e., lower *K*_m_) and/or produce more exoenzymes per unit biomass (i.e., higher *V*_max/MBC_) in a proportion that favors higher *V*_max/MBC_/*K*_m_ ratios (i.e., CE_MBC_). As the *K*_m_-values of BG and LAP were relatively invariable between 30 and 90 cm, their lower CE_MBC_ at depths below 60 cm was mainly driven by a decline in *V*_max/MBC_, suggesting that it was constrained by lower production of enzymes per unit biomass below that depth rather than higher *K*_m_. On the other hand, enzymes may optimize *k*_cat_ in adaptation to environmental pressures (e.g., temperature) at the expense of *K*_m_, leading to conformational adaptations that reduce active site binding, which result in higher *K*_m_ and suboptimal catalytic efficiencies (Struvay and Feller, 2012). Therefore, a lower CE_MBC_ in deeper soils driven by lower *V*_max/MBC_ may also reflect enzymes with lower *k*_cat_, as a result of trade-offs with *K*_m_, rather than lower enzyme production. The contrasting lack of variation in CE_MBC_ of AP with depth, regardless of individual variation in *V*_max/MBC_ and *K*_m_, might have resulted from different factors and interactions related to variation in relative P availability and demand, and in regulation of AP expression. Alternatively, P may be primarily acquired from minerals rather than organic compounds (Alori et al., 2017), and thus the CE_MBC_ of AP alone does not directly reflect P demand. It should also be noted that variation in enzyme and substrate diffusion through the soil matrix, or their stabilization through mineral-organic interactions, can lead to differences in substrate availability and enzyme accessibility. For example, lower diffusivity or higher adsorption of enzymes or substrates to soil particles could select for enzymes with higher affinities and prompt higher enzyme production per cell. Conversely, stabilization of active enzymes can increase their longevity and effectiveness over time (Burns et al., 2013), which could alleviate the selective pressure on their catalytic efficiency, as we observed in deeper soils.

Our results suggest that microbial communities in deep subsoils encode exoenzymes with intrinsically lower *k*_cat_, and/or produce less enzymes per cell than those in surface soils, leading to a lower emergent CE_MBC_. The expectation that *V*_max/MBC_ and CE_MBC_ would increase with depth assumes that microbiomes have largely redundant metabolic and elemental demands, and thus that exoenzymes are optimized to provide nutrients in proportion to the size and demands of the community, as a function of nutrient availability (Allison et al., 2011). However, several studies have shown that microbiomes change markedly with soil depth (Fierer et al., 2003b; Hansel et al., 2008; Hartmann et al., 2009; Eilers et al., 2012; Jiao et al., 2018; Liu et al., 2019), with deep soils harboring less diverse and functionally distinct organisms (Brewer et al., 2019; Diamond et al., 2019; Yan et al., 2019; Dove et al., 2021). Life strategies that prioritize cellular maintenance over fast growth and maximal resource exploitation, and properties such as utilization of alternative substrates, storage compound production, and ability to sporulate or undergo dormancy may all affect exoenzyme properties, and possibly contribute to relax selective pressures on their kinetics (Ho et al., 2017; Ramin and Allison, 2019). Dove et al. (2021) have shown that deep soil microbes at our site have lower growth rates and lower carbon use efficiency than those at the surface, reflecting a lower nutrient demand and greater investment on cellular maintenance that may underlie the lower *V*_max/MBC_ and CE_MBC_ we observed. Moreover, the declining substrate availability with depth is expected to decrease the return on exoenzyme investment and favor alternative metabolic strategies that do not rely primarily on depolymerization of complex organic matter. Dove et al. (2021) have indeed shown that microbiomes in these deep soils have lower potential to degrade complex carbohydrates, similar to those in other soils (Diamond et al., 2019). Conversely, it has been shown that deep soil communities are enriched in organisms that can metabolize one-carbon (C_1_) and other low-molecular weight C compounds, and use inorganic N forms as energy sources (Brewer et al., 2019; Diamond et al., 2019), as well as in taxa that comprise mainly chemoautotrophs (Cao et al., 2012; Eilers et al., 2012; Turner et al., 2017; Brewer et al., 2019; Diamond et al., 2019). Therefore, a smaller fraction of organisms relying on exoenzymes in deep soils is also likely to contribute to a lower emergent CE_MBC_ due to both lower overall biomass-specific enzyme production and lower competition for enzyme products within the community. Nevertheless, it should be noted that, while microbial biomass largely dominates living matter in mineral and usually dry soils with low root density, such as these, we cannot entirely exclude minor contributions of enzymes produced by plants and soil fauna to the activities measured.

### Relative Kinetics Between Exoenzymes Vary With Depth, Suggesting Variation in Nutrient Demands

As expected, *V*_max_ differed significantly between all three enzymes at every depth, reflecting fundamental differences in relative nutrient demand, as well as possible differences in enzyme properties and regulation. According to ecoenzymatic stoichiometry, ratios between *V*_max_ of hydrolytic enzymes involved in acquisition of C, N or P reflect the relative demand in these elements in relation to their availability, and thus the equilibrium between microbial biomass and SOM stoichiometry (Sinsabaugh and Shah, 2012). Here, both *V*_max_ and CE ratios between C- and N-acquiring enzymes (*V*_max_^BG:LAP^ and CE^BG:LAP^, respectively) were relatively constant through the soil profile, despite suggestive increases at lower depths, which were consistent with the trend in soil C:N ratio (Hicks Pries et al., 2018) and ratios between dissolved C and N pools (Supplementary Figure 4C). At the same time, *V*_max_ and CE ratios between BG:AP and LAP:AP decreased with depth, suggesting an increasing demand for P relative to either C or N, similar to previous observations of ratios between activities of C- and P-acquiring enzymes in both temperate and tropical soils (Stone et al., 2014; Loeppmann et al., 2016a). Alternatively, it has been shown that soil microbes can use phosphorylated compounds primarily as a C source (Heuck et al., 2015), and thus higher *V*_max_ and CE of AP may rather indicate higher C demand in the absence of more favorable C sources in deep soils, as previous suggested (Stone et al., 2014). It should be noted, however, that depolymerization of organic matter and nutrient acquisition involves also other enzymes, and therefore the enzyme investigated here may not fully represent C, N or P demand and availability (Sinsabaugh and Shah, 2012).

### Exoenzyme Temperature Sensitivity Is Similar Through the Soil Profile Across Enzyme Types

Our results confirmed the general expectation that activity of BG, LAP and AP is stimulated by increasing temperatures up to optimal temperatures above those typically observed in moderate environments, with *Q*_10_-values for *V*_max_ and activation energies within the ranges typically observed for soil exoenzymes (Trasar-Cepeda et al., 2007; Brzostek and Finzi, 2012; German et al., 2012; Stone et al., 2012; Steinweg et al., 2013; Razavi et al., 2015, 2016; Nottingham et al., 2016). However, despite few suggestive trends, temperature sensitivity did not vary significantly with depth for any enzyme or kinetic property, based on either *Q*_10_, following a linear Arrhenius model, or *T*_opt_, TS_max_ and Δ*C*_p_^‡^ of *V*_max_ estimated by the non-linear MMRT model. We could not determine unambiguously whether the response of *V*_max_ to temperature was best explained by Arrhenius or MMRT models, although comparisons suggested that the latter generally fit the data better. Moreover, positive temperature response rates of *V*_max_ declined at higher temperatures in most cases (i.e., above TS_max_ = 31.63 ± 1.98°C, up to *T*_opt_ = 65.19 ± 3.74°C, followed by a negative response), as observed by other studies (Alster et al., 2016a,^2018^), further indicating that MMRT represented a more realistic temperature response behavior. On the other hand, this also indicated that the linear models captured the temperature response better under lower temperatures within the native temperature range, before response rates slowed towards *T*_opt_, similar to what has been observed by Alster et al. (2016a). The uniform temperature sensitivity observed here contradicted our initial hypothesis that exoenzymes in deeper soils are more sensitive to temperature changes as a result of microbial adaptation to lower and narrower temperature ranges (Schipper et al., 2014), as observed at our site. Previous studies have observed that exoenzymes from colder soil environments tend to be more sensitive to temperature (Koch et al., 2007; Brzostek and Finzi, 2012), as well as in some subsoils in relation to surface soils (Steinweg et al., 2013). Therefore, we expected that *Q*_10_-values and activation energies would increase with depth, whereas either *T*_opt_, Δ*C*_p_^‡^, or both, would decrease. Under MMRT’s Optimum-Driven hypothesis, the more frequent lower temperatures in deeper soils could select for enzymes with lower *T*_opt_, regardless of their Δ*C*_p_^‡^ (Alster et al., 2020). Conversely, the Thermal Breadth hypothesis postulates that enzymes subject to large temperature ranges have less negative Δ*C*_p_^‡^ (i.e., flatter temperature response curves) but not necessarily different *T*_opt_, and thus the narrower temperature ranges in deeper soils would lead to more negative Δ*C*_p_^‡^ (Alster et al., 2020). In turn, the Enzyme Rigidity hypothesis predicts that cold-adapted enzymes have more negative Δ*C*_p_^‡^ due to their lower rigidity, which would lead to a decline in Δ*C*_p_^‡^, and consequently *T*_opt_, with depth, following selection of enzymes adapted to lower temperatures (Alster et al., 2020). The uniform temperature sensitivity of all enzymes through the soil profile may instead reflect a convergence of enzyme *T*_opt_ towards the similar MATs across depths at our site (10.4–11.5°C), despite different temperature ranges. On the other hand, the low MATs of our soils appear to contradict the relatively high Δ*C*_p_^‡^ observed, compared to values previously reported (Alster et al., 2016a,^2018^), which are expected to reflect a high enzyme rigidity typical of warm-adapted enzymes. It is possible that enzyme Δ*C*_p_^‡^ in our soils are mainly driven by their wide temperature ranges, despite their narrowing with depth, leading to selection of enzymes with less negative Δ*C*_p_^‡^, and thus able to maintain more constant activity rates under varying temperatures (Alster et al., 2020). Moreover, reactions potentially involving a diverse isozyme pool, such as those measured here, reflect the summation of the temperature response curves of those enzymes, and thus are also expected to have a less negative Δ*C*_p_^‡^ (Alster et al., 2018).

Our results are consistent with the uniform temperature sensitivity (apparent *Q*_10_) of *in situ* soil respiration over the top meter of soil previously observed at this site (Hicks Pries et al., 2017). This suggests that SOM depolymerization by exoenzymes may be closely linked to the response of total soil respiration to temperature, likely by modulating the contribution of microbial heterotrophic metabolism. In turn, *K*_m_ was relatively insensitive to temperature, with mean *Q*_10_-values around 1 across depths and enzymes. This suggests that enzyme affinities may be biochemically constrained to prevent being affected by temperature fluctuations, as previously suggested (Allison et al., 2018). *T*_opt_ of *V*_max_ (65.19 ± 3.74°C), based on the MMRT model, was much higher than natural soil temperatures, whereas TS_max_ (31.63 ± 1.98°C) was just above the temperature maximum in surface soils (29°C at 5 cm), but substantially higher than those at lower depths (19°C at 30 cm and 16°C at 100 cm), or mean annual temperatures (MAT) over the upper meter of soil (10.4–11.5°C). Similar high *T*_opt_ estimates based on MMRT have been generally observed for microbial exoenzyme activities and complex metabolic processes in soils (Schipper et al., 2014), and for soil bacterial isolates (Alster et al., 2016a). This is consistent with the fact that the thermal stability and optimal catalytic temperature of enzymes from mesophilic organisms tend to be higher than that of their native environment (Engqvist, 2018). While persistent warming is expected to generally induce higher enzyme activity through the whole soil profile, a uniform TS_max_ may, however, result in variable net annual temperature responses at different depths due to their different temperature ranges, duration of different temperature regimes, and seasonal variation, regardless of similar MATs. How these factors may interact in response to sustained long-term warming, and their outcomes, will depend on the degree of thermal adaptability of exoenzymes through changes in microbial community composition, and expression of isozymes with different properties (Wallenstein et al., 2011; Bradford, 2013). It should be noted, however, that we cannot completely rule out the possibility that the spatial variability of some temperature sensitivity estimates might have precluded detection of robust differences between depths. Such variability is a common limitation of measurements of emergent responses of complex biological systems, especially due to sample-specific variables that cannot be accounted for with the methods currently available. These challenges emphasize the need for novel approaches that allow a more reproducible assessment of such processes across spatiotemporal scales.

### Different Exoenzymes Have Overall Similar Temperature Sensitivities but Temperature Can Affect Their Relative Kinetics at Discrete Depths

The magnitude of all temperature sensitivity parameters was remarkably similar between enzymes through the soil profile, although *Q*_10_-values of *V*_max_ and *K*_m_ have been frequently shown to vary between co-occurring soil enzymes (Wallenstein et al., 2011). Likewise, *T*_opt_, TS_max_ and Δ*C*_p_^‡^ can vary substantially between enzymes (Alster et al., 2016a). Nevertheless, we did observe a significantly lower *Q*_10_ of *K*_m_ and consequently significantly higher *Q*_10_ of CE of LAP in the upper 10 cm, relative to the other enzymes. The fact that the mean *Q*_10_ of *K*_m_ of LAP was below 1 (*Q*_10_ = 0.83), while those of BG and AP were not (*Q*_10_ = 1.30 and 1.18, respectively), suggested that the affinity of LAP was positively stimulated by higher temperatures at this depth (i.e., *K*_m_ decreased), or that those of BG and AP were negatively affected. As the *Q*_10_ of *V*_max_ did not differ significantly between any enzyme, this led to a significantly higher positive temperature response of the CE of LAP. This likely contributed to the significant negative effect of higher temperatures on the CE^BG:LAP^ ratio, and shows that temperature can directly affect the relative catalytic efficiencies between C- and N-acquiring enzymes. This was consistent with previous observations suggesting that kinetic responses to temperature may vary among enzyme types, leading to changes in relative cycling of different nutrients (Allison et al., 2018). Moreover, the significant interaction between depth and temperature on CE^BG:LAP^ ratios confirmed that their variation with depth was dependent on temperature, possibly reflecting the higher CE *Q*_10_ of LAP in the upper 10 cm. Higher temperatures also had a significant positive effect on *V*_max_^BG:AP^ and *V*_max_^LAP:AP^ ratios, indicating that AP was generally less stimulated by higher temperature than BG or LAP. As enzyme assays at different temperatures were performed with the same soil preparations per depth and incubated over short periods, these relative differences in *V*_max_ likely reflected a direct effect of temperature on enzyme *k*_cat_, independently of enzyme concentration. Despite the uniform temperature sensitivity of all kinetic parameters of individual enzymes through the soil profile, these results show that temperature can affects differently the intrinsic kinetic properties (i.e., *K*_m_ and *k*_cat_) of distinct enzymes in a depth-dependent manner, presumably without active microbial regulation.

## Conclusion

Kinetic and thermal properties of exoenzymes are fundamental components of complex trait spaces that allow microbes to thrive under variable nutrient availability and temperature regimes, as well as other interacting selective pressures (Allison et al., 2011; Sinsabaugh and Shah, 2012; Sinsabaugh et al., 2014; Ho et al., 2017; Ramin and Allison, 2019; Malik et al., 2020). Our results indicate a strong variation in exoenzyme kinetics through the soil profile. We propose that this may reflect variation in substrate availability, differences in exoenzyme production, and/or expression of distinct isozymes. These possibilities, however, require further investigation. Moreover, we show that the temperature sensitivity of specific kinetic properties is remarkably similar through the soil profile and between enzymes, although it can, at least in some cases, differ between enzymes at discrete depths. This suggested that temperature may directly affect relative substrate depolymerization and nutrient acquisition potential, effectively decoupling enzyme relative activities from other regulatory factors, such as nutrient demand and substrate availability. Although microbial trait spaces are not static, as microbiomes adapt to changing conditions, they are likely to constrain both immediate responses and the trajectory of longer-term responses to environmental changes (Conant et al., 2011; Bradford, 2013; Xu et al., 2021). Therefore, it is essential to identify and validate key microbial traits and their environmental constraints in order to build a mechanistic understanding that can be generalized across spatiotemporal scales, and combine theory, measurements, and models to improve the representation of microbial processes in Earth system models (Wieder et al., 2015; Blankinship et al., 2018). Together, our results improve the mechanistic understanding of microbial processes driving SOM dynamics as a function of soil depth and temperature, and provide new directions towards improved representation of key microbial traits in depth-resolved biogeochemical models.

## Data Availability Statement

The original contributions presented in the study are included in the article/Supplementary Material, further inquiries can be directed to the corresponding author/s.

## Author Contributions

RA, MM, and EB conceived and designed the study. RA, IC, GM, and EB collected soil samples. RA, IC, HS, and BW performed soil chemical analyses and enzyme assays. RA analyzed and interpreted the data with support from IC, GM, MM, and EB. RA wrote the manuscript with input from MM, MT, and EB. All authors read and reviewed the manuscript.

## Conflict of Interest

The authors declare that the research was conducted in the absence of any commercial or financial relationships that could be construed as a potential conflict of interest.

## Publisher’s Note

All claims expressed in this article are solely those of the authors and do not necessarily represent those of their affiliated organizations, or those of the publisher, the editors and the reviewers. Any product that may be evaluated in this article, or claim that may be made by its manufacturer, is not guaranteed or endorsed by the publisher.
