# Supplementary material for: Kinetic Properties of Microbial Exoenzymes Vary With Soil Depth but Have Similar Temperature Sensitivities Through the Soil Profile
Source: Front Microbiol. 2021 Nov 30;12:735282. doi: 10.3389/fmicb.2021.735282 (PMC8669745; doi:10.3389/fmicb.2021.735282)
Supplement: Supplementary file 1 [file Data_Sheet_1.PDF]

Supplementary Material

Supplementary Results and Discussion

Comparison of temperature sensitivity based on Arrhenius and Macromolecular Rate Theory models

The temperature sensitivity of enzyme V_{max} was determined using a linear Arrhenius model over the full temperature range tested (4-50°C, $n = 6$) (Figure S2) and a realistic *in situ* soil range (4-35°C, $n = 5$) (Figure 5), as well as the non-linear MMRT model over the full temperature range (4-50°C, $n = 6$). Enzyme thermal traits analyzed here and their definitions are indicated in Table 2. The great majority of individual MMRT models (n = 54) approached the expected unimodal behavior, whose highest point represents T_{opt} (Figure 6). Despite the relatively high adjusted R^2 values of the linear Arrhenius models over the full temperature range (0.81 ± 0.02, mean ± se) (Table S6), there was a general decline in the response rate of V_{max} between the two highest temperatures consistent with MMRT predictions, even when T_{opt} was not reached (Figure 6). The temperature response of V_{max} in four of the 54 individual models did not fit the assumptions of MMRT, resulting in biologically implausible T_{opt} and TS_{max} estimates for these soils, namely negative values due to an upward concave response (AP at 10-20 cm and BG at 50-60 cm, replicate 3), or values above 200°C due to approximately linear responses (BG at 60-70 cm, replicate 1; and BG at 10-20 cm, replicate 3) (Figure 6, Table S7). These model estimates were thus excluded from further analyses. Comparisons between alternative Arrhenius and MMRT models based on the Akaike Information Criterion (AIC), corrected AIC (AICc) and Bayesian Information Criterion (BIC) standard indices indicated that, in general, their model fit did not differ substantially (see also Supplementary Results). Nevertheless, overall model comparisons consistently favored MMRT by marginal differences, which further suggested that MMRT provided a more realistic representation of the temperature response of enzyme V_{max}. AICc consistently over-penalized the model with the highest number of parameters ($K = 3$; MMRT) or the lowest number of temperature data-points ($n = 5$; Arrhenius over 4-35°C), resulting in disproportionally high ΔAICc values in relation to the model with the lowest number of parameters and highest number of temperatures ($K = 2$ and $n = 6$; Arrhenius over 4-50°C) (Table S6). This was likely due to the low overall ratio between sample size (i.e., number of temperatures) and number of parameters (n/K ratio) in our models, and the additional correction for this ratio used by AICc, which put a disproportional high weight on very small differences of just one parameter or data-point. Moreover, the number of parameters in our three-parameter MMRT model is effectively the same as that in the Arrhenius model ($K = 2$), due to the interdependence between enthalpy and entropy parameters (Alster et al., 2020). The less stringent AIC index values were more consistent with the observed data trends, adjusted R^2 values of the linear models, and differences in n/K ratio (Figures 5-6, Table S6). Absolute AIC values indicated that MMRT had the highest fit for 50% of the datasets, followed by Arrhenius over 4-50°C and then Arrhenius over 4-35°C. These results were mirrored by the BIC index, although this index has a greater penalty factor for low n/K ratios than AIC (Tables S6). Nevertheless, ΔAIC values and AIC relative likelihoods showed substantial support for most alternative models, according to the guidelines by (Burnham and Anderson, 2004), indicating that, in general, the fit of alternative Arrhenius and MMRT models did not differ substantially (Tables S6). The few ΔAIC exceptions to this trend indicated that only 17% of the Arrhenius models over 4-50°C and 7% of those over 4-35°C had substantially worse

fit than their two alternative models for the same dataset, whereas MMRT model fits were never substantially worse than their Arrhenius counterparts.

References

Alster, C. J., von Fischer, J. C., Allison, S. D., and Treseder, K. K. (2020). Embracing a new paradigm for temperature sensitivity of soil microbes. *Glob. Chang. Biol.* 26, 3221–3229. doi:10.1111/gcb.15053.

Burnham, K. P., and Anderson, D. R. (2004). Multimodel inference: Understanding AIC and BIC in model selection. *Sociol. Methods Res.* 33, 261–304. doi:10.1177/0049124104268644.

Supplementary Tables

Table S1. Soil and microbial biomass chemistry (additional spreadsheet included in Supplementary Materials).

Table S2. Michaelis-Menten kinetic parameters (additional spreadsheet included in Supplementary Materials).

Table S3. Two-way fixed effects ANOVA of kinetic parameters with enzyme type and temperature as independent factors, per depth. Differences were considered significant at p < 0.05 (values in bold font).

		$V_{max/ds}$			$V_{max/MBC}$			K_m			CE_{ds}			CE_{MBC}		
**Depth (cm)**	**Factor**	**Df**	***F* value**	***p* value**	**Df**	***F* value**	***p* value**	**Df**	***F* value**	***p* value**	**Df**	***F* value**	***p* value**	**Df**	***F* value**	***p* value**
00-10	Enzyme	2	359.48	**< 0.05**	2	360.20	**< 0.05**	2	246.78	**< 0.05**	2	923.88	**< 0.05**	2	2774.82	**< 0.05**
	Temperature	5	30.50	**< 0.05**	5	30.56	**< 0.05**	5	1.98	0.11	5	26.31	**< 0.05**	5	79.01	**< 0.05**
	Enzyme × Temperature	10	0.22	0.99	10	0.22	0.99	10	5.04	**< 0.05**	10	2.53	**< 0.05**	10	7.61	**< 0.05**
10-20	Enzyme	2	61.57	**< 0.05**	2	57.03	**< 0.05**	2	57.62	**< 0.05**	2	121.82	**< 0.05**	2	28.54	**< 0.05**
	Temperature	5	6.43	**< 0.05**	5	6.43	**< 0.05**	5	0.36	0.88	5	4.17	**< 0.05**	5	5.40	**< 0.05**
	Enzyme × Temperature	10	0.32	0.97	10	0.95	0.50	10	0.49	0.885	10	0.17	1.00	10	1.37	0.24
30-40	Enzyme	2	114.38	**< 0.05**	2	298.98	**< 0.05**	2	128.68	**< 0.05**	2	526.70	**< 0.05**	2	697.14	**< 0.05**
	Temperature	5	10.24	**< 0.05**	5	26.77	**< 0.05**	5	0.18	0.97	5	15.34	**< 0.05**	5	20.31	**< 0.05**
	Enzyme × Temperature	10	0.13	1.00	10	0.33	0.97	10	1.07	0.41	10	0.72	0.70	10	0.95	0.50
50-60	Enzyme	2	120.86	**< 0.05**	2	207.69	**< 0.05**	2	168.22	**< 0.05**	2	302.36	**< 0.05**	2	447.57	**< 0.05**
	Temperature	5	11.44	**< 0.05**	5	20.46	**< 0.05**	5	1.00	0.43	5	9.49	**< 0.05**	5	14.53	**< 0.05**
	Enzyme × Temperature	10	0.39	0.94	10	0.59	0.81	10	0.78	0.65	10	0.63	0.78	10	0.88	0.56
60-70	Enzyme	2	76.55	**< 0.05**	2	173.35	**< 0.05**	2	85.51	**< 0.05**	2	136.38	**< 0.05**	2	276.20	**< 0.05**
	Temperature	5	5.54	**< 0.05**	5	12.58	**< 0.05**	5	1.18	0.34	5	4.67	**< 0.05**	5	9.58	**< 0.05**
	Enzyme × Temperature	10	0.31	0.98	10	0.68	0.73	10	0.87	0.57	10	0.10	1.00	10	0.14	1.00
80-90	Enzyme	2	145.88	**< 0.05**	2	163.42	**< 0.05**	2	30.63	**< 0.05**	2	308.30	**< 0.05**	2	345.72	**< 0.05**
	Temperature	5	8.13	**< 0.05**	5	9.50	**< 0.05**	5	0.96	0.45	5	10.74	**< 0.05**	5	12.34	**< 0.05**
	Enzyme × Temperature	10	0.39	0.94	10	0.43	0.92	10	0.96	0.50	10	0.47	0.90	10	0.48	0.89

Table S4. Variation in Michaelis-Menten kinetic parameters between enzymes per depth. Different colors and letters indicate significant differences ($p < 0.05$), based on Tukey's tests after two-way ANOVA per depth (Table S3).

Depth (cm)	Enzyme	$V_{max/ds}$	$V_{max/MBC}$	K_m	CE_{ds}	CE_{MBC}
00-10	BG	b	b	b	a	b
	LAP	c	c	a	b	c
	AP	a	a	b	a	a
10-20	BG	b	b	c	a	a
	LAP	c	c	a	b	b
	AP	a	a	b	a	a
30-40	BG	b	b	c	a	a
	LAP	c	c	a	b	b
	AP	a	a	b	a	a
50-60	BG	b	b	c	b	b
	LAP	c	c	a	c	c
	AP	a	a	b	a	a
60-70	BG	b	b	b	b	b
	LAP	c	c	a	c	c
	AP	a	a	b	a	a
80-90	BG	b	b	b	b	b
	LAP	c	c	a	c	c
	AP	a	a	b	a	a

Table S5. Two-way fixed effects ANOVA of ratios between kinetic parameters of BG, LAP and AP, with depth and temperature as independent factors. Differences were considered significant at $p < 0.05$ (values in bold font).

	$V_{max}^{BG:LAP}$			$V_{max}^{BG:AP}$			$V_{max}^{LAP:AP}$		
**Factor**	**Df**	***F* value**	***p* value**	**Df**	***F* value**	***p* value**	**Df**	***F* value**	***p* value**
Depth	5	6.38	**< 0.05**	5	24.81	**< 0.05**	5	9.00	**< 0.05**
Temperature	5	1.20	0.32	5	5.30	**< 0.05**	5	2.41	**< 0.05**
Depth × Temperature	25	0.35	1.00	25	0.82	0.70	25	0.35	1.00

	$K_m^{BG:LAP}$			$K_m^{BG:AP}$			$K_m^{LAP:AP}$		
**Factor**	**Df**	***F* value**	***p* value**	**Df**	***F* value**	***p* value**	**Df**	***F* value**	***p* value**
Depth	5	5.78	**< 0.05**	5	11.32	**< 0.05**	5	1.21	0.314
Temperature	5	2.14	0.07	5	2.32	0.05	5	0.50	0.77
Depth × Temperature	25	1.27	0.22	25	0.76	0.77	25	0.76	0.78

	$CE^{BG:LAP}$			$CE^{BG:AP}$			$CE^{LAP:AP}$		
**Factor**	**Df**	***F* value**	***p* value**	**Df**	***F* value**	***p* value**	**Df**	***F* value**	***p* value**
Depth	5	4.24	**< 0.05**	5	15.91	**< 0.05**	5	9.95	**< 0.05**
Temperature	5	9.72	**< 0.05**	5	0.63	0.68	5	2.21	0.06
Depth × Temperature	25	1.85	**< 0.05**	25	0.59	0.93	25	0.26	1.00

Table S6. Fit indices for Arrhenius models over 4-50°C and 4-35°C, and MMRT model over 4-50°C (additional spreadsheet included in Supplementary Materials).

Table S7. Q_{10} coefficients and activation energy (E_a) estimated over 4-50°C and 4-35°C, and MMRT parameter estimates over 4-50°C from all individual models. Different background colors indicate different models or different temperature ranges. Values excluded from further analyses are indicated in red (see main text) (additional spreadsheet included in Supplementary Materials).

Table S8. Mean Q_{10} coefficients and activation energy (E_a) estimated over 4-50°C and 4-35°C, and MMRT parameter estimates over 4-50°C, per enzyme and depth. Different background colors indicate different models or different temperature ranges (additional spreadsheet included in Supplementary Materials).

Table S9. One-way ANOVA of temperature sensitivity parameters between enzymes per depth. Q_{10} coefficients were calculated over 4-35°C, and the MMRT model parameters T_{opt}, TS_{max}, and $\Delta C_p^{\ddagger}$ over 4-50°C. Differences were considered significant at $p < 0.05$ (values in bold font).

	V_{max} Q_{10}			K_m Q_{10}			CE Q_{10}			T_{opt}			TS_{max}			$\Delta C_p^{\ddagger}$		
**Depth (cm)**	**Df**	***F* value**	***p* value**	**Df**	***F* value**	***p* value**	**Df**	***F* value**	***p* value**	**Df**	***F* value**	***p* value**	**Df**	***F* value**	***p* value**	**Df**	***F* value**	***p* value**
00-10	2	0.28	0.76	2	14.58	**< 0.05**	2	35.90	**< 0.05**	2	1.33	0.33	2	1.68	0.26	2	0.72	0.52
10-20	2	0.75	0.51	2	3.59	0.09	2	4.68	0.06	2	0.12	0.89	2	0.41	0.69	2	0.05	0.95
30-40	2	4.65	0.06	2	5.22	0.05	2	5.13	0.05	2	0.69	0.54	2	1.14	0.38	2	< 0.01	1.00
50-60	2	3.80	0.09	2	0.39	0.69	2	1.98	0.22	2	0.30	0.75	2	0.19	0.83	2	1.10	0.40
60-70	2	6.38	**0.03**	2	1.60	0.28	2	0.05	0.95	2	4.00	0.09	2	2.00	0.23	2	3.66	0.11
80-90	2	1.16	0.38	2	0.21	0.82	2	0.12	0.89	2	2.38	0.17	2	1.98	0.22	2	1.09	0.40

Table S10. Variation in temperature sensitivity between enzymes per depth. Only temperature sensitivity parameters and depths where they varied significantly between enzymes are shown. Q_{10} values were calculated over 4-35°C. Different colors and letters indicate significant differences (p < 0.05) based on Tukey's tests after one-way ANOVA per depth (Table S9).

Depth (cm)	Enzyme	V_{max} Q_{10}	K_m Q_{10}	CE Q_{10}
00-10	BG	a	a	b
	LAP	a	b	a
	AP	a	a	b
60-70	BG	ab	a	a
	LAP	a	a	a
	AP	b	a	a

Supplementary Figures

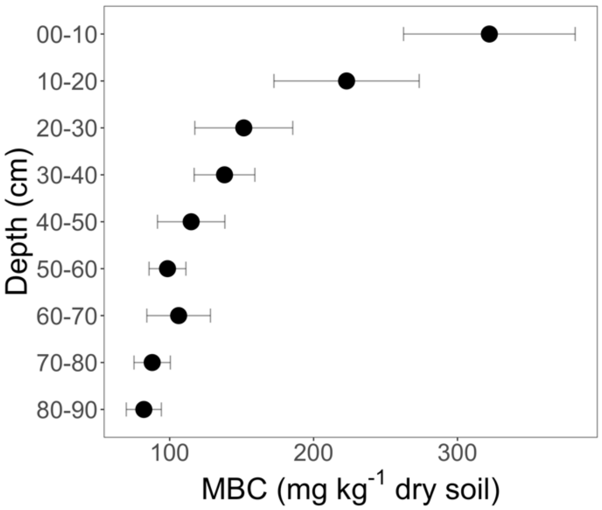

Figure S1. Microbial biomass carbon (MBC) concentration through the soil profile. Error bars indicate the standard error of the mean (n = 3).

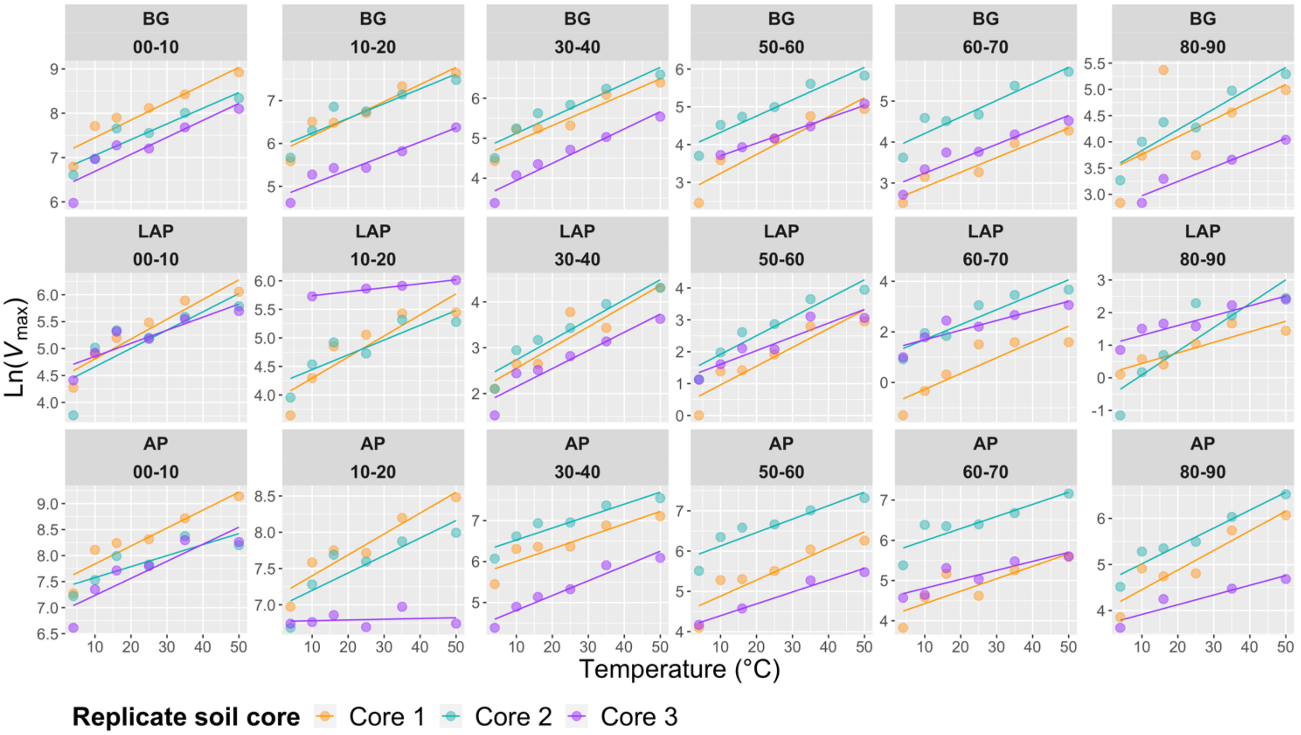

Figure S2. Arrhenius models of V_{max} over five temperatures from 4 to 50°C per enzyme, depth and replicate core sample.

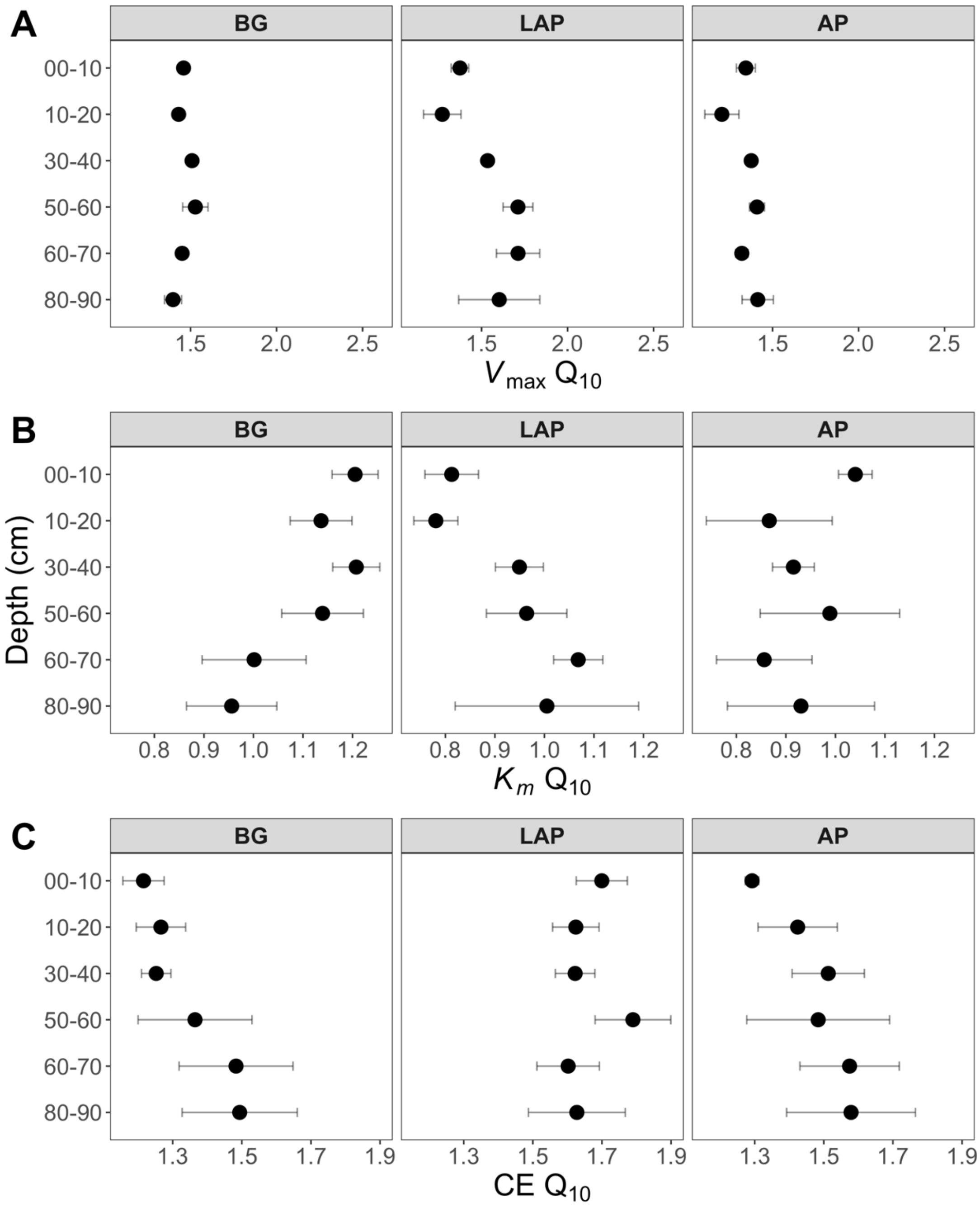

Figure S3. Q10 coefficients of MM kinetic parameters over six temperatures from 4 to 50°C at different depths. (A) Vmax, (B) Km, and (C) CE. Differences between depths are not significant ($p > 0.05$), based on ANOVA per enzyme (Table 2). Error bars represent the standard error of the mean ($n = 3$).

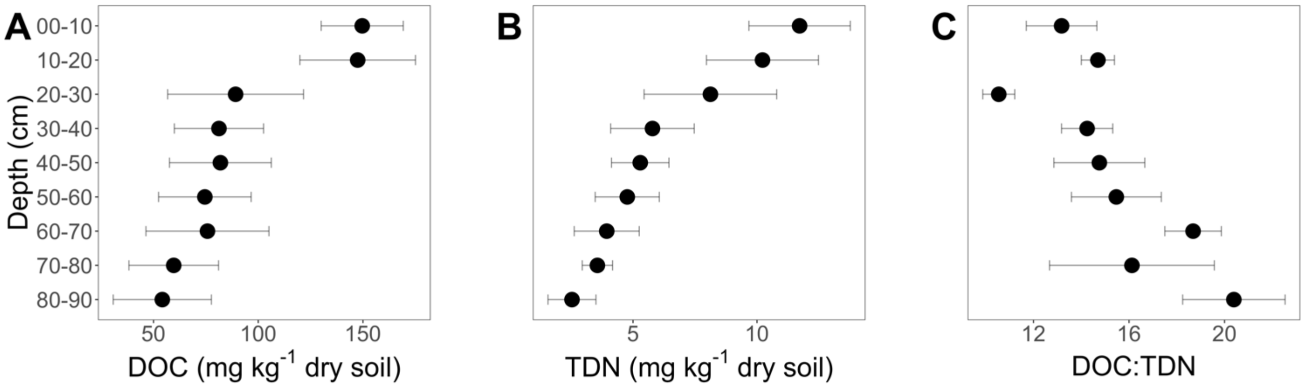

Figure S4. Soil (A) dissolved organic carbon (DOC), (B) total dissolved nitrogen (TDN), and (C) DOC:TDN ratio. Error bars indicate the standard error of the mean (n = 3).
