# Supplementary material for: Kinetic Properties of Microbial Exoenzymes Vary With Soil Depth but Have Similar Temperature Sensitivities Through the Soil Profile
Source: Front Microbiol. 2021 Nov 30;12:735282. doi: 10.3389/fmicb.2021.735282 (PMC8669745; doi:10.3389/fmicb.2021.735282)
Supplement: Supplementary file 2 [file Data_Sheet_2.zip › Supplementary_Materials_File_Descriptions.pdf]

Supplementary Materials – File descriptions

enzymes_code.zip	
enzymeAssay_dataParsingCode.zip	Contains 31 R notebooks with annotated code to parse the raw measurement data in each of the corresponding 31 enzyme assay output files contained in "dataRaw.zip > enzymeAssay_dataRaw.zip". The code parses the raw plate assay data, including control and calibration measurements and calculates calibrated enzyme net fluorescence values. Data is parsed based on the assay plate layout shown in "dataRaw.zip > enzymeAssay_metadata.zip > BL.OP.201907_Enzymes_AssayPlateLayouts.xlsx". Data is labeled using the assay metadata file "dataRaw.zip > enzymeAssay_metadata.zip > BL.OP.201907_Enzymes_Metadata.txt".
enzymes_dataAnalysis.Rmd	R notebook with code to (1) collect and merge the calibrated enzyme net fluorescence data output of each of the 31 R notebook files in "enzymeAssay_dataParsingCode.zip"; (2) quality-check, correct and select data or downstream analysis; (3) calculate enzyme activity rates, and model enzyme kinetics and temperature sensitivity; (4) perform all statistical analyses and plot results. Data labeling and calculations require the files "BL.OP.201907_Enzymes_Incubation_Time_Selection.txt"; "BL.OP.201907_Enzymes_Metadata.txt"; "sampleID_to_IGSN.csv"; and "BL.OP.201907_Soil.Chemistry.txt"; contained in "dataRaw.zip > enzymeAssay_metadata.zip".
soilChem_dataAnalysis.Rmd	R notebook with annotated code to calculate and plot soil and microbial biomass chemistry data from input file "dataRaw.zip > soilChemistry_dataRaw.zip > BL.OP.201907_Soil.Chemistry.Raw.tsv".

enzymes_dataProcessed.zip	
enzymeActivity_corr.csv	10,368 corrected measurements of potential activity of the exoenzymes beta-glucosidase, leucine/leucyl aminopeptidase, and acid phosphatase in 18 soil samples collected from six depths in each of three replicate soil cores after incubation with each of eight substrate concentrations and under each of six temperatures. Values include quadruplicate measurements under each combination of conditions.
enzymeKinetics_corr.csv	313 corrected values of each of five Michaelis-Menten kinetic parameters of the exoenzymes beta-glucosidase, leucine/leucyl aminopeptidase, and acid phosphatase in 18 soil samples collected from six depths in each of three replicate soil cores, after incubation under each of six temperatures. Michaelis-Menten kinetics were modeled over quadruplicate measurements of enzyme activity under each of the combinations of conditions.

	Individual enzyme activity measurements are available in file "enzymeActivity_corr.csv". Parameters: maximum reaction velocity (Vmax; on dry soil and microbial biomass C basis); half-saturation/Michaelis constant (Km); and catalytic efficiency (CE; on dry soil and microbial biomass C basis).
enzymeKinetics_corr_means.csv	108 mean values and respective standard deviations and standard errors of each of five Michaelis-Menten kinetic parameters of the exoenzymes beta-glucosidase, leucine/leucyl aminopeptidase, and acid phosphatase in triplicate soil samples collected from each of six soil depths, after incubation under each of six temperatures. Individual replicate values are available in file "enzymeKinetics_corr.csv". Michaelis-Menten kinetics were modeled over quadruplicate measurements of enzyme activity under each of the combinations of conditions (file "enzymeActivity_corr.csv"). Parameters: maximum reaction velocity (Vmax; on dry soil and microbial biomass C basis); half-saturation/Michaelis constant (Km); and catalytic efficiency (CE; on dry soil and microbial biomass C basis).
enzymeTempSens_corr.csv	54 corrected values of each of multiple temperature sensitivity parameters of the exoenzymes beta-glucosidase, leucine/leucyl aminopeptidase, and acid phosphatase in 18 soil samples collected from six depths in each of three replicate soil cores. Parameters: activation energy and Q10 indices of Vmax, Km, and catalytic efficiency (linear Arrhenius model); temperature optimum; point of maximum temperature sensitivity; and change in heat capacity (non-linear Macromolecular Rate Theory model). Arrhenius parameters and Q10 were modeled over five temperatures between 4-35 degree Celsius and six temperatures between 4-50 degree Celsius; Macromolecular Rate Theory parameters were modeled over only six temperatures between 4-50 degree Celsius.
enzymeTempSens_corr_means.csv	18 mean values, and respective standard deviations and standard errors, of each of multiple temperature sensitivity parameters of the exoenzymes beta-glucosidase, leucine/leucyl aminopeptidase, and acid phosphatase in triplicate soil samples collected from each of six soil depths. Individual replicate values are available in file "enzymeTempSens_corr.csv". Parameters: activation energy and Q10 indices of Vmax; Km; and catalytic efficiency (linear Arrhenius model); temperature optimum; point of maximum temperature sensitivity; and change in heat capacity (non-linear Macromolecular Rate Theory model). Arrhenius parameters and Q10 were modeled over five temperatures between 4-35 degree Celsius and six temperatures between 4-50 degree Celsius; Macromolecular Rate Theory parameters were modeled over only six temperatures between 4-50 degree Celsius.
soilChemistry.csv	Soil and microbial biomass chemistry of 27 soil samples collected from nine depths in each of three replicate soil cores. Properties measured: GWC; DOC; TDN; MBC; MBN; and DOC:TDN and MBC:MBN ratios.

enzymes_dataRaw.zip	
enzymeAssay_dataRaw.zip	Contains 31 raw spectrophotometer output files with fluorescence measurements of enzyme plate assays. Each file contains fluorescence measurements from 12x 96-well plate assays for 3 enzymes; including controls and calibration standards; incubated at one of 6 incubation temperatures and for one of 2-3 incubation periods (see Experimental Design Table 1 in file "enzymeTraits_design_results_tables.xlsx"). Input files for data parsing R notebooks in "code.zip > enzymeAssay_dataParsingCode.zip".
enzymeAssay_metadata.zip	Contains 6 metadata files required to parse; label and calculate soil and microbial biomass chemistry; and enzyme activity data. Input for R notebooks in "code.zip".
soilChemistry_dataRaw.zip	Contains 1 file with raw data on soil and microbil biomass chemistry. Input for R notebook in "code.zip > soilChem_dataAnalysis.Rmd".
